# Supplementary material for: Differentiable sampling of molecular geometries with uncertainty-based adversarial attacks
Source: Nat Commun. 2021 Aug 24;12:5104. doi: 10.1038/s41467-021-25342-8 (PMC8384857; doi:10.1038/s41467-021-25342-8)
Supplement: Supplementary file 1 — Supplementary Information [file 41467_2021_25342_MOESM1_ESM.pdf]

Supplementary Information for: Differentiable sampling of molecular geometries with uncertainty-based adversarial attacks

Daniel Schwalbe-Koda,*) Aik Rui Tan,*) and Rafael Gómez-Bombarelli†)

Department of Materials Science and Engineering, Massachusetts Institute of Technology, Cambridge, MA 02139

(Dated: 31 July 2021)

*)D.S.-K. and A.R.T. contributed equally to this work

†)Electronic mail: rafagb@mit.edu

SUPPLEMENTARY NOTES

Supplementary Note 1. One-dimensional double well potential

To exemplify the training of the NN potential and the adversarial attacks, we analyze a simple, one-dimensional (1D) double well potential. At first, we employ a symmetrical potential described by

$$E(x) = 5x^4 - 10x^2. \tag{1}$$

Following the same methods employed for the 2D double well potential, we train the NN committee on ground truth data generated for this potential. Only data points (r, E, F) with $E < -3.5$ are included in the train set. Fig. 1a shows the resulting prediction and train statistics for this system. The uncertainty in the energy increases for the barrier between the two wells, since no training data points are found in that region. While that is mostly the case for the variance of the forces, these also suffer from a drop around $r = 0$. This indicates that the networks agree that the force should go to zero near the point $r = 0$ to make the two wells coincide.

A statistical analysis of the uncertainties is shown in Fig. 1b. By taking data points with energy below zero which are not in the training set, we construct a test set where the NN ensemble is in the extrapolation regime. Then, we compare the variances of energies and forces for points in both the train and test sets. Overall, the distribution of variances in forces of the train set overlaps less with the test set distribution than their energy variance counterparts. This becomes clearer when the percentiles of the train (test) set are taken with respect to the variances of the test (train) distribution, as shown in Fig. 1c. In the case of force variances, most of the test set points are above the 70th percentile of the variances of the train set. Conversely, uncertainties of the train set are under the 20th percentile of the distribution of test set variances. This is not true for energy variances, where the test set has points with percentiles as low as 20th with respect to the train set.

Empirically, it can be seen that adopting the classification threshold t (see Eq. (6) of the main text) as the 80th percentile of the forces variance from the train set is a reasonable choice. Fig. 1d shows the relationship between the mean absolute error (MAE) of energy and force predictions and the location of the 80th percentile of the variances. Whereas higher

errors are correlated to higher variances, a high variance does not necessarily imply a high error. This is often the case of undersampled regions where the interpolating power of the NN potential is able to perform good predictions. Moreover, errors are more pronounced for variances above the 80th percentile threshold for forces, illustrating its classification power for epistemic error.

Using the variance in forces σ_F^2 as the uncertainty metric, we construct the adversarial loss $\mathcal{L}_{\text{adv}}$ using Eq. (11) from the main paper, as shown in Fig. 2. In particular, Fig. 2d illustrates that upon a reasonable choice of temperature, transition states and points beyond the training set are favorably sampled by the adversarial attack.

To demonstrate the ability of adversarial attacks and active learning loops to efficiently explore the phase space, we repeat Fig. 2a of the main text for the 1D well (Fig. 3) by imposing an offset between the two wells,

$$E(x) = 5x^4 - 10x^2 + 1.5x. \tag{2}$$

After the successive application of adversarial attacks, the well centered at $x = 1$ is discovered and sampled until the uncertainty in the region becomes below the 80th percentile of the force variance. The choice of temperature often prevents the adversarial loss from going towards infinity as $r \to \pm\infty$ unless the predicted energy becomes negative in these directions (generations 2 and 3 of Fig. 3). Sampling the points $r \to \pm\infty$ is avoided by using a fixed number of steps for optimizing δ, which essentially limits how much the attack can travel.

If the energy uncertainty σ_E^2 is employed to create the adversarial loss $\mathcal{L}_{\text{adv}}$ instead of the force uncertainty, the exploration of the phase space is not performed as efficiently. Fig. 4 exemplifies an active learning loop for the 1D double well described by Eq. (2). Due to the force matching strategy when training the NN potential, the uncertainty outside of the training set is not necessarily higher than that within the training domains. As such, the adversarial attack on the uncertainty does not necessarily reward sampling new regions of the phase space, preventing the active learning loop from proceeding adequately every generation.

Supplementary Note 2. Combining collective variable attacks and all-atom translations

In addition to performing adversarial attacks on predefined collective variables (CVs), we can also sample new geometries by applying small distortions (δ) to the positions of all atoms, thus creating new geometries not seen in the training data. After training each generation of NN committee on the original molecular dynamics data (see Methods for NN training details), 700 training configurations from the training data are randomly chosen as seed geometries for the adversarial attacks. To ensure that physically meaningful configurations are obtained from the sampling, the normalized temperature kT of the adversarial loss is set to 3 kcal/mol. The attacks were performed for 80 epochs using the Adam optimizer with a learning rate of 5×10^{-3}. This all-atom (AA) adversarial attack strategy was performed for 3 generations. Although the AA attack strategy allows the NN committee to sample various high-energy configurations, the phase space is not well explored, as CVs of attacked geometries are similar to the original seed CVs. In fact, the robustness of NN potential does not increase even when the size of the training data increases by around 20 % (Fig. 14).

To sufficiently explore the configuration space, we coupled 3 generations of AA attacks after 7 generations of CV attacks (CV + AA) (see Fig. 15). The CV adversarial attack follows the same procedure employed in the main paper (see Methods). After 7 generations of CV attacks, 3 generations of AA attacks with the procedure described above were performed. For each generation, 500 adversarial attacks were sampled. Half of the seed geometries was randomly selected from CV adversarial attacks while the other half was taken from the molecular dynamics data. While AA attacks alone fail to improve robustness of NN potentials, CV + AA attacks were able to yield much longer stable MD trajectories (Fig. 14). This suggests that CV attacks are necessary to capture collective dynamics such as bond rotations which are not easily explored via translation-based adversarial attacks. CV attacks allow the sampled configurations to access geometries outside the low-energy bounds. On the other hand, translation-based adversarial attacks supplement the diverse vibrational space offered by the increasing number of atoms in the system.

Supplementary Note 3. Adversarial attacks using ANI-1x models

To exemplify the use of adversarial sampling with different architectures and data sets, we performed adversarial attacks using ANI-1x models on three molecules from the ANI-1x dataset: methane (CH_4), ammonia (NH_3), and water (H_2O), in addition to the alanine dipeptide system studied in this work. 42 methane, 36 ammonia and 22 water molecules were randomly selected from the ANI-1x data set, and 100 alanine dipeptide configurations were randomly selected from the dataset from Section III.C of main paper. The ANI-1x dataset was obtained from the TorchANI v2.2 GitHub repository[1]. The normalized temperature was kT set to 0.7 kcal/mol and adversarial attacks were performed for 70 steps for methane, ammonia and water, and 100 steps for alanine dipeptide using the Adam optimizer at a learning rate of 5×10^{-5}. The NN ensemble consists of 8 ANI-1x models, pretrained on the ANI-1x data set. The evolution of the maximum standard deviation of atomic forces across models (max force std) as a function of steps is shown in Fig. 17a. Even though the max force std does not increase monotonically when all seeds are analyzed at once, adversarial attacks continuously attempt to push molecules towards configurations of higher uncertainty. Within 100 steps, many configurations of high RMSD are obtained (Fig. 17b). Since thermodynamic likelihoods of molecules are taken into account, total energies of molecules rarely exceed 100 kcal/mol above the ground state energy, even at relatively high RMSDs (Fig. 18c). In some cases, the attacked geometries have relatively low RMSD (< 0.05) compared to the training set (see Table 1). Intuitively, attack geometries with higher RMSD could improve the robustness of neural network potentials, although there may exist a trade-off between accuracy and transferability towards high energy regions of the configuration space (see Section III.C of the main paper). In many cases, however, NN potentials fail even within training domain of data. Hence, the adversarial sampling strategy samples configurations most likely to confuse the models. This task would otherwise be difficult to sample without a differentiable uncertainty metric, even when the sampled structures are geometrically similar to the training domain due to the non-linearity of NN predictions.

To compare the force uncertainties across different molecules, the distributions of the relative force std and max force std as a function of energy per atom are shown in Figs

[1] Available at `https://github.com/aiqm/torchani`

18a,b. The relative force std is calculated according to

$$\sigma_F^{(\mathrm{rel})} = \max \frac{\sigma_{F,i}}{|\mathbf{F}_i|}, \tag{3}$$

with $i = \arg\max \sigma_{F,i}$.

Since methane, ammonia and water are much smaller than alanine dipeptide, a smaller number of adversarial steps is usually required to push the geometries outside the training domains of the neural network models. However, we have also attempted adversarial attacks for small molecules with a larger number of steps to verify if higher uncertainty configurations could be obtained without breaking the molecules (see Figs. 19 and 20). Interestingly, at a high normalized temperature and with just 30 extra steps, more distorted geometries can be obtained from the seed configurations, all of which have high force uncertainty. However, the energy of these new configurations is much higher compared to the training set, some of which exceed 10 kcal/mol/atom. This indicates that obtaining geometries outside of the ANI-1x training set requires pushing towards much higher energy configurations.

I. SUPPLEMENTARY FIGURES

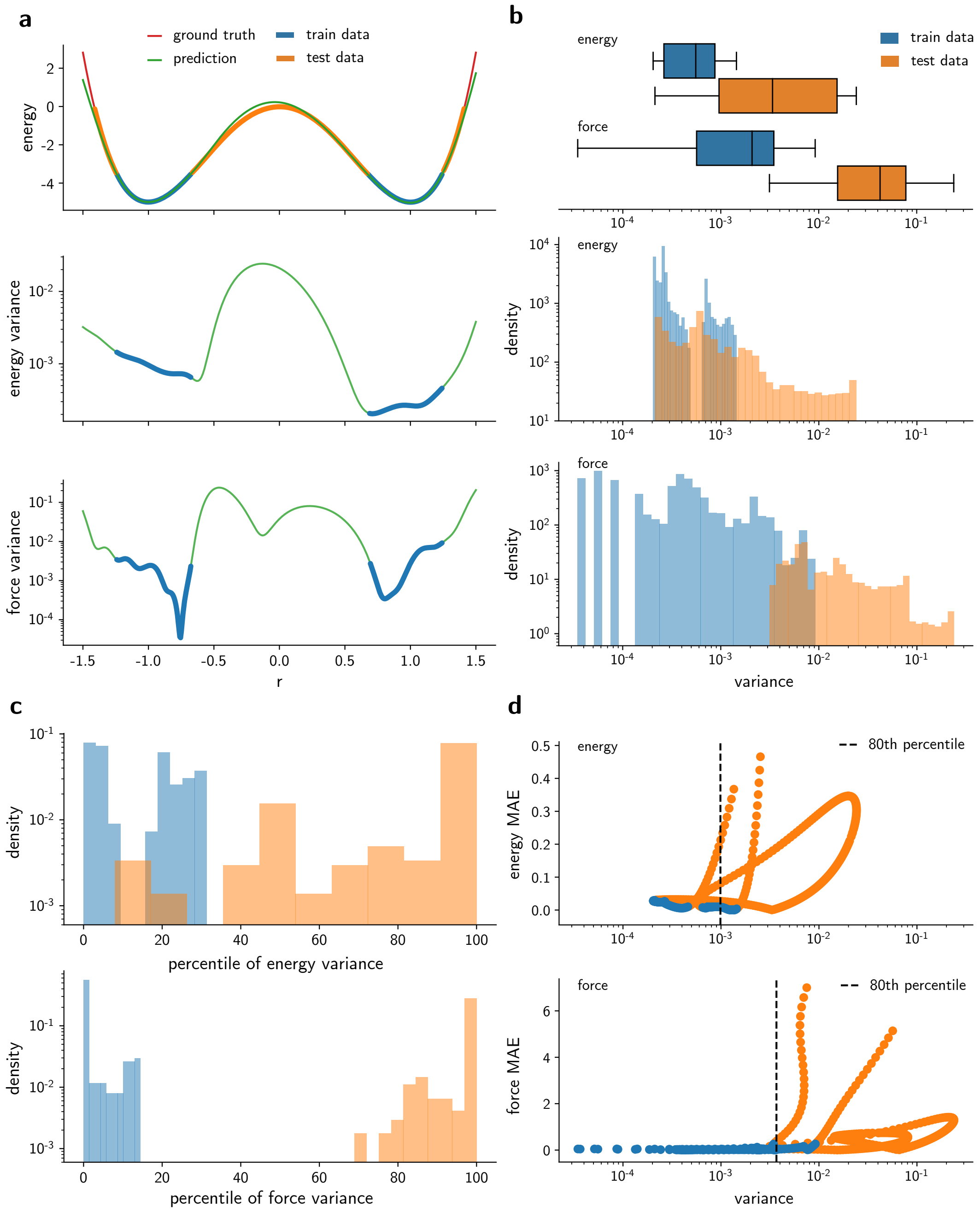

Supplementary Figure 1. Example of training statistics for the 1D double well potential. **a**, Predictions of energy, energy variance and force variance for five networks trained on the data shown in blue. **b**, Statistics of the models on the train/test data shown in **a**. The vertical line is the median, boxes are the interquartile region, and whiskers represent the range of the distribution. **c**, Distribution of energy and force variances. A smaller overlap between variances of test and train data is found for forces, as opposed to energy predictions. **d**, Relationship between the mean absolute error (MAE) of energies and forces and their corresponding variances. MAE is computed with respect to the ground truth values.

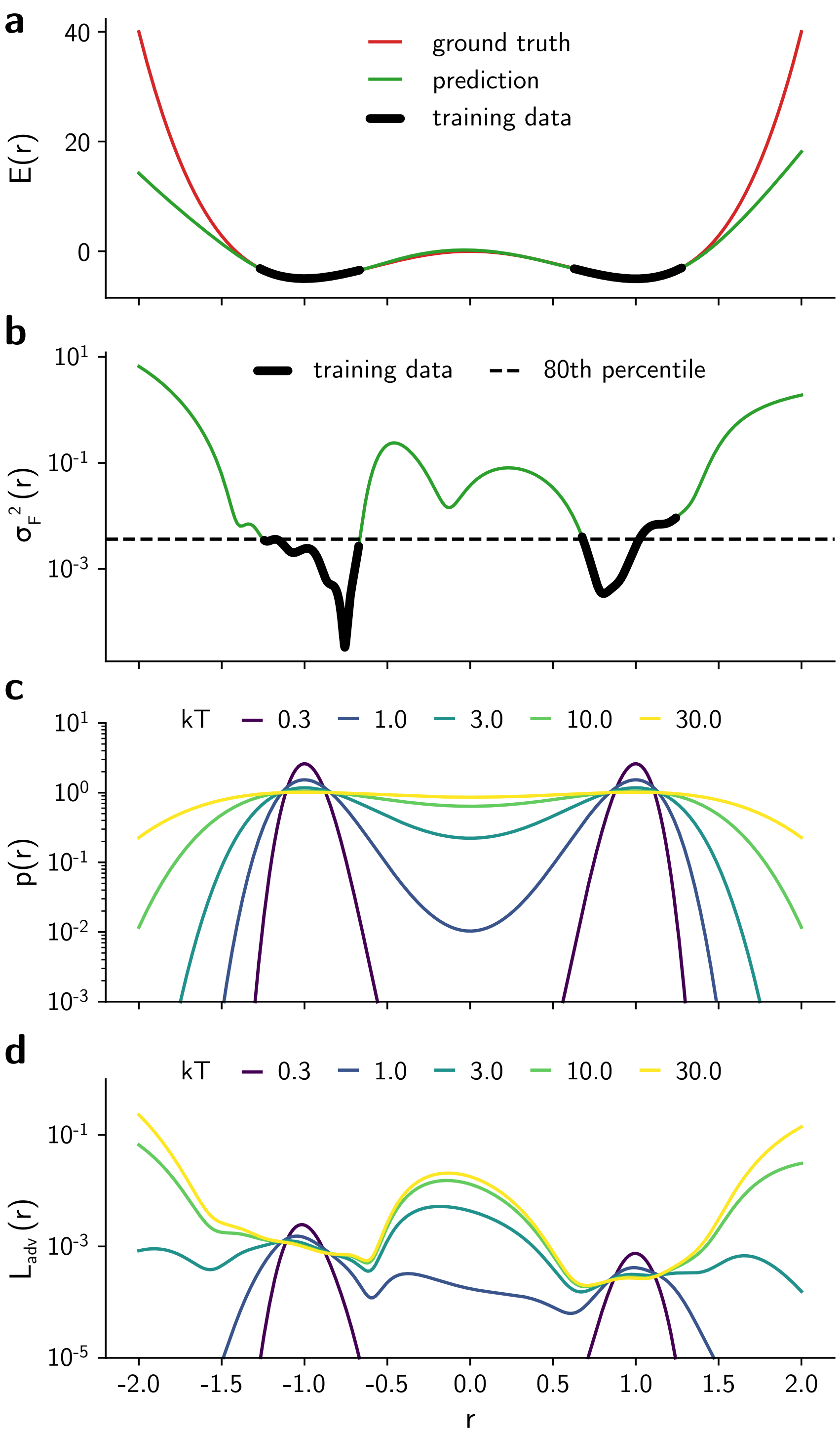

Supplementary Figure 2. Example of loss function for the example shown in Fig. 1. Predictions of **a**, mean energy and **b**, force variance for the given range of positions r. **c**, Boltzmann probability constructed for the ground truth potential. A higher value of kT allows higher energy states to be sampled with higher probability. **d**, Adversarial loss constructed for the neural network potential. For $kT > 3$, the adversarial attack rewards sampling the transition state of the double well potential.

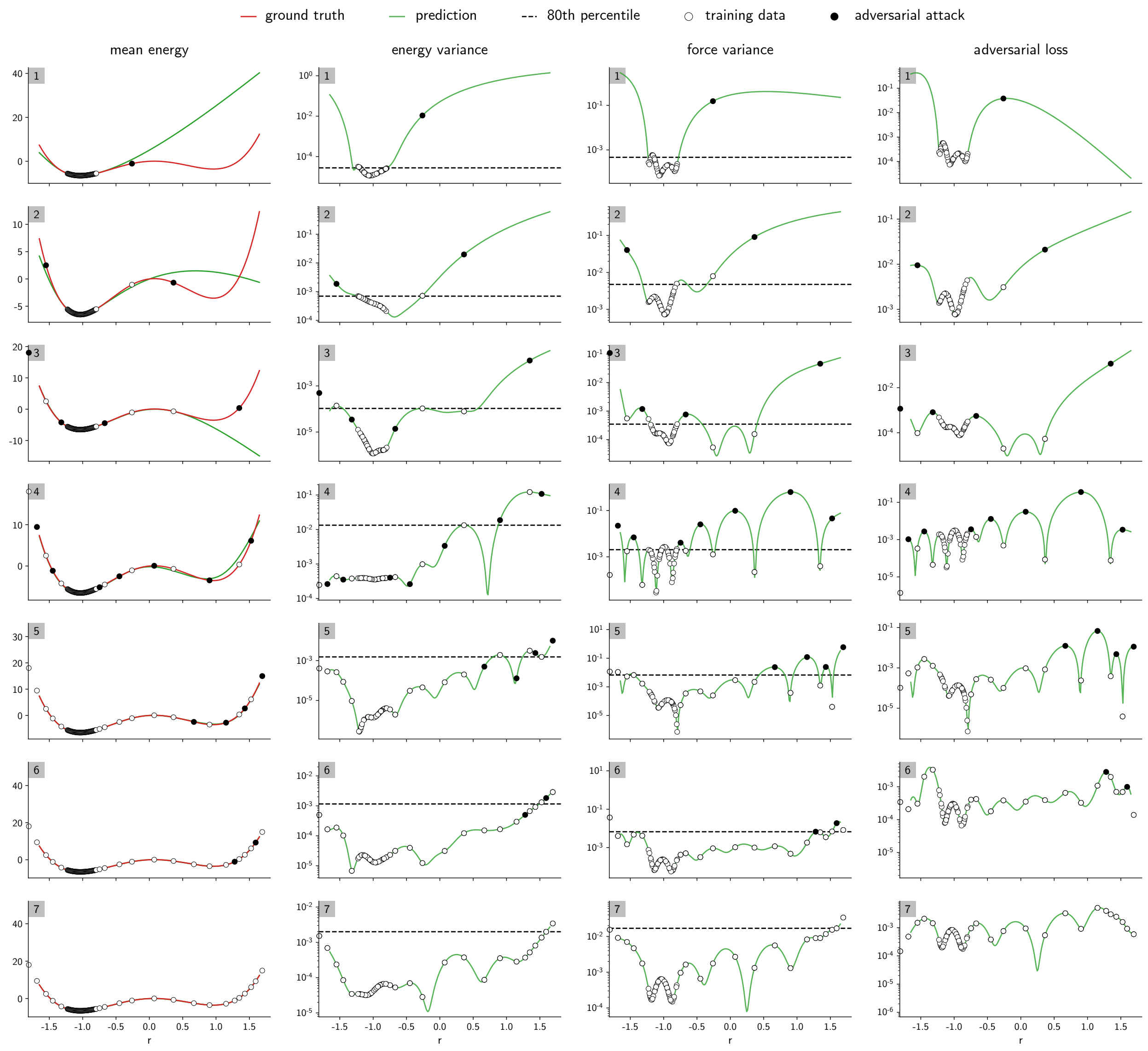

Supplementary Figure 3. Evolution of mean energy, energy and force variance, and adversarial loss for a one dimensional double well potential. The number of the generation is shown on the top left corner of each plot. The normalized temperature for the adversarial loss is 5. The dashed line is the 80th percentile of the variance of the training data, and is often useful for deduplication.

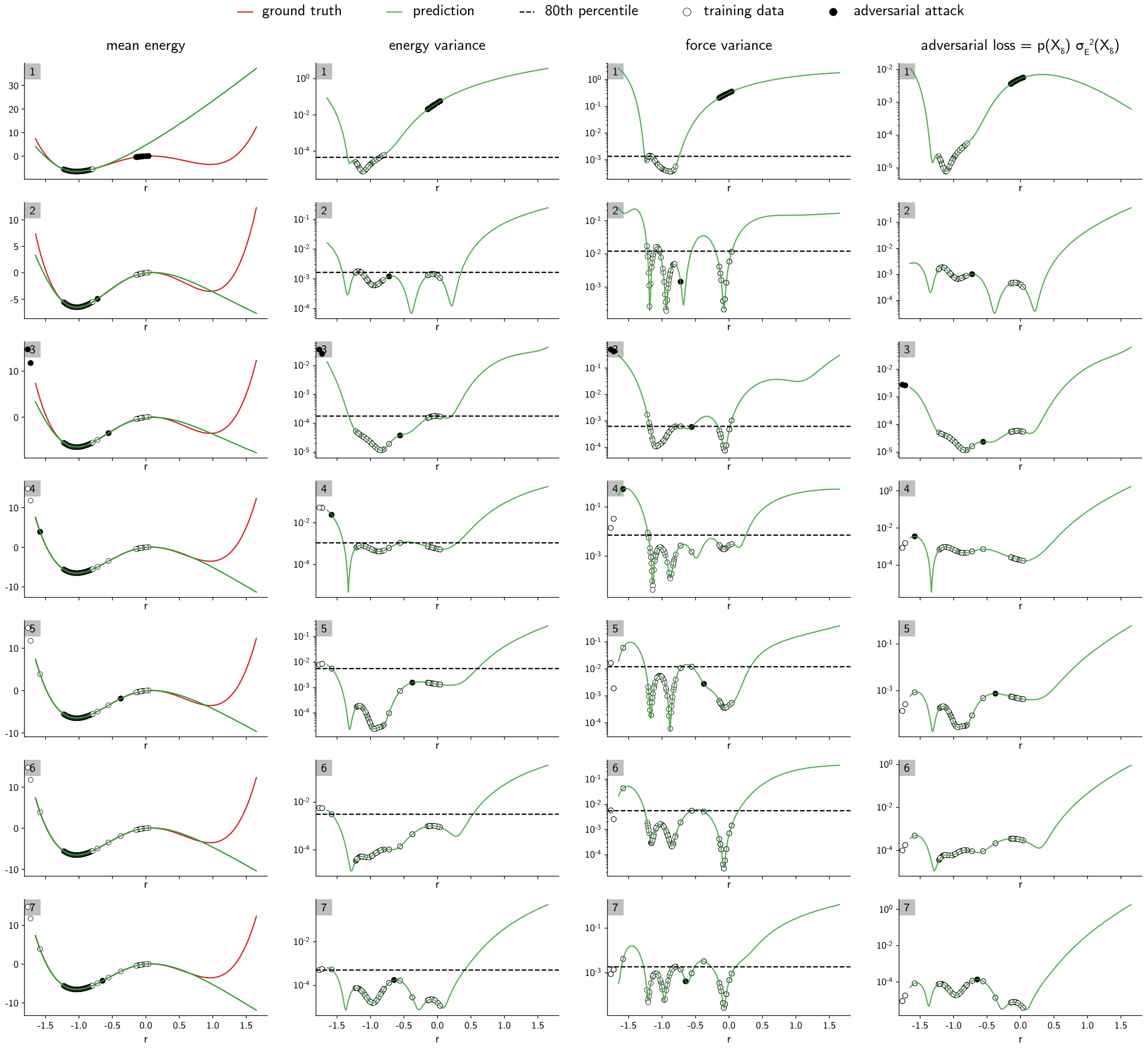

Supplementary Figure 4. Evolution of mean energy, energy and force variance, and modified adversarial loss for a one dimensional double well potential. The adversarial loss from Eq. (13) of the main paper was modified to use σ_E^2 instead of σ_F^2. Due to the force matching approach, the energy uncertainty does not allow the space to be adequately explored. The number of the generation is shown on the top left corner of each plot. The normalized temperature for the adversarial loss is 5. The dashed line is the 80th percentile of the variance of the training data.

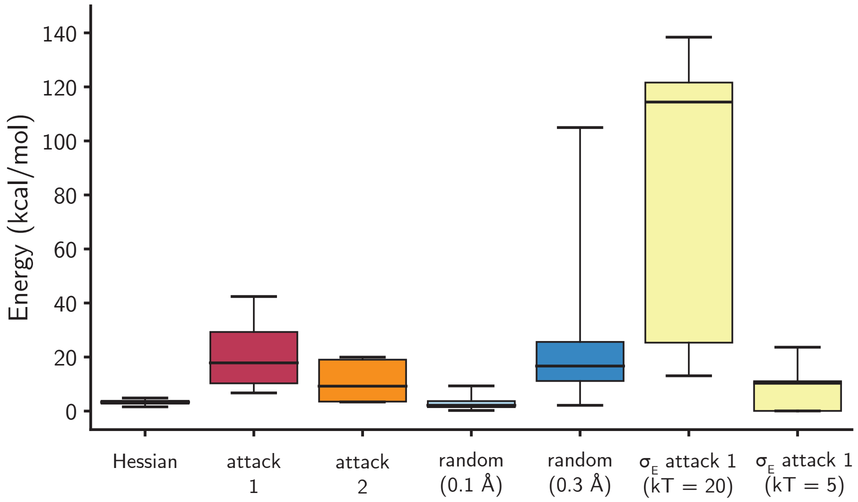

Supplementary Figure 5. Distribution of DFT energies for conformations of an ammonia molecule sampled with different methods, including adversarial attacks performed using the energy uncertainty (σ_E^2). The horizontal line is the median, the box is the interquartile region and the whiskers span the range of the distribution.

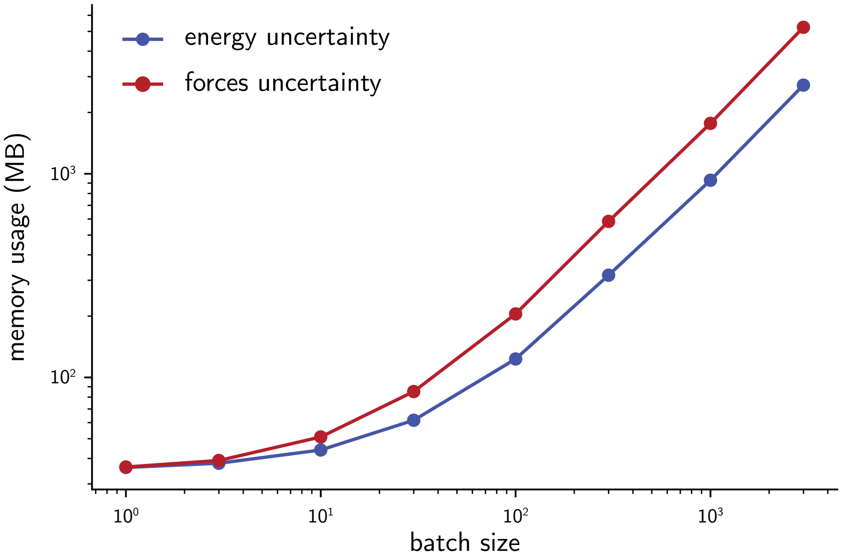

Supplementary Figure 6. Total GPU memory used by 5 SchNet models, a batch of ammonia molecules, and its computational graph when the uncertainty in energy (blue) and forces (red) are calculated. The cached GPU memory is not taken into consideration in this plot.

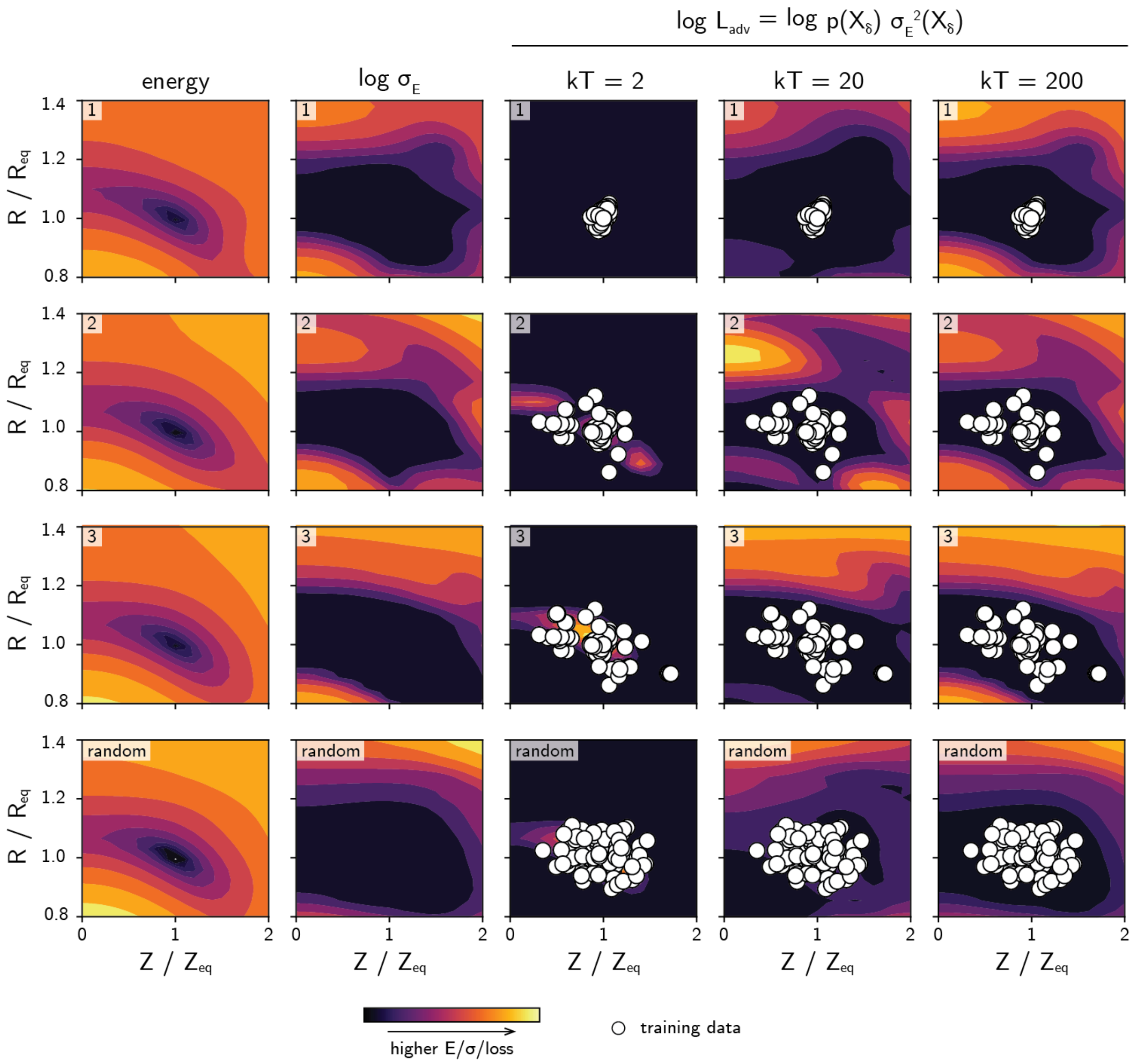

Supplementary Figure 7. Relationship between the projected PES, energy uncertainty and adversarial loss for ammonia. When the energy variance is used for the adversarial loss function, smaller contrasts between regions within and outside of the training set typically lead to a worse exploration of the phase space when compared to an adversarial loss based on forces uncertainty (see Fig. 8). The number of the generation is shown on the top left corner of each plot. The variables R and Z are defined in Fig. 3 of the main text. kT is given in kcal/mol.

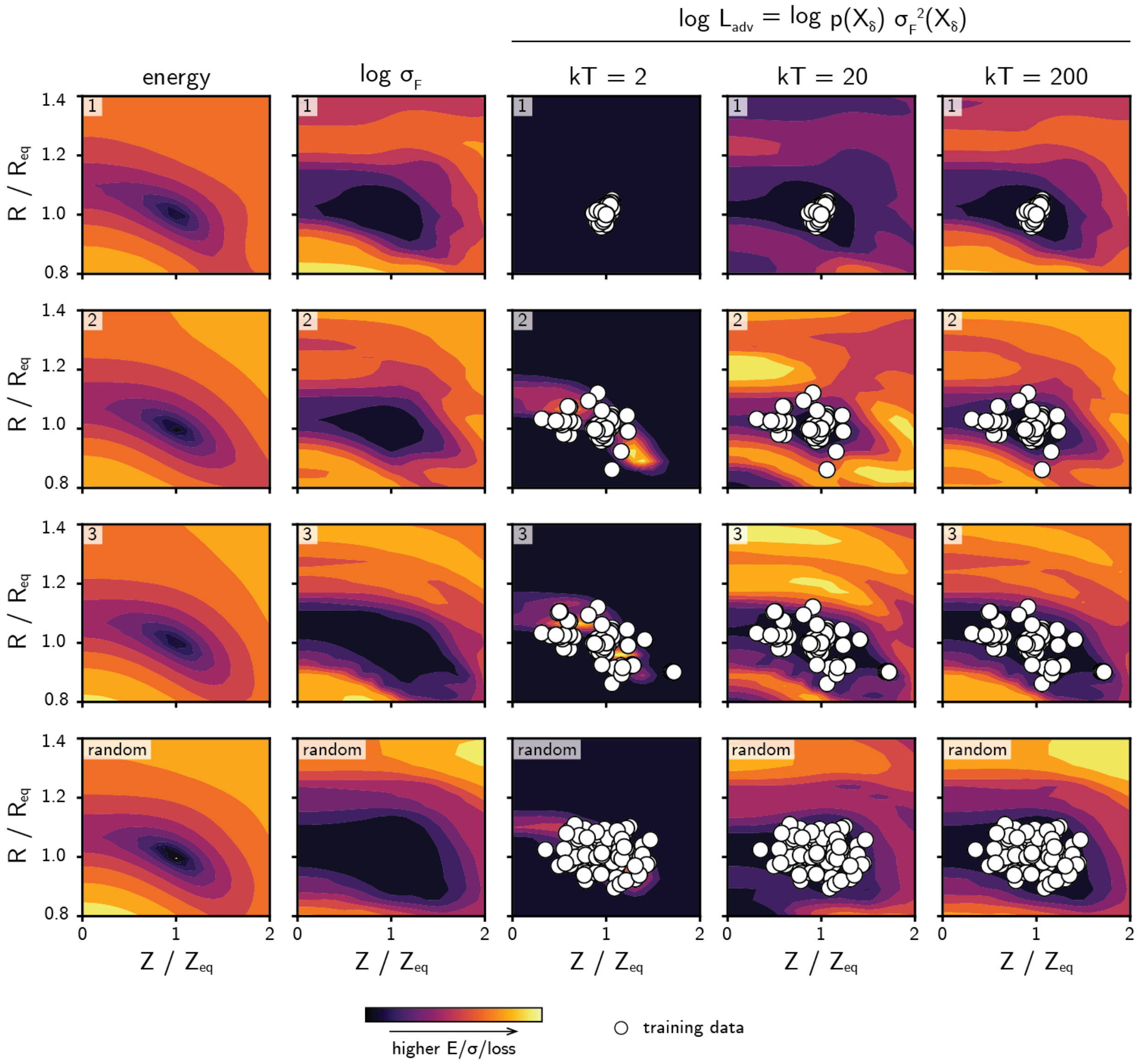

Supplementary Figure 8. Relationship between the projected PES, energy uncertainty and adversarial loss for ammonia. When the force variance is used for the adversarial loss function, a higher contrast between regions within and outside of the training set favor an informed exploration of the phase space when compared to an adversarial loss based on energy uncertainty (see Fig. 7). The number of the generation is shown on the top left corner of each plot. The variables R and Z are defined in Fig. 3 of the main text. kT is given in kcal/mol.

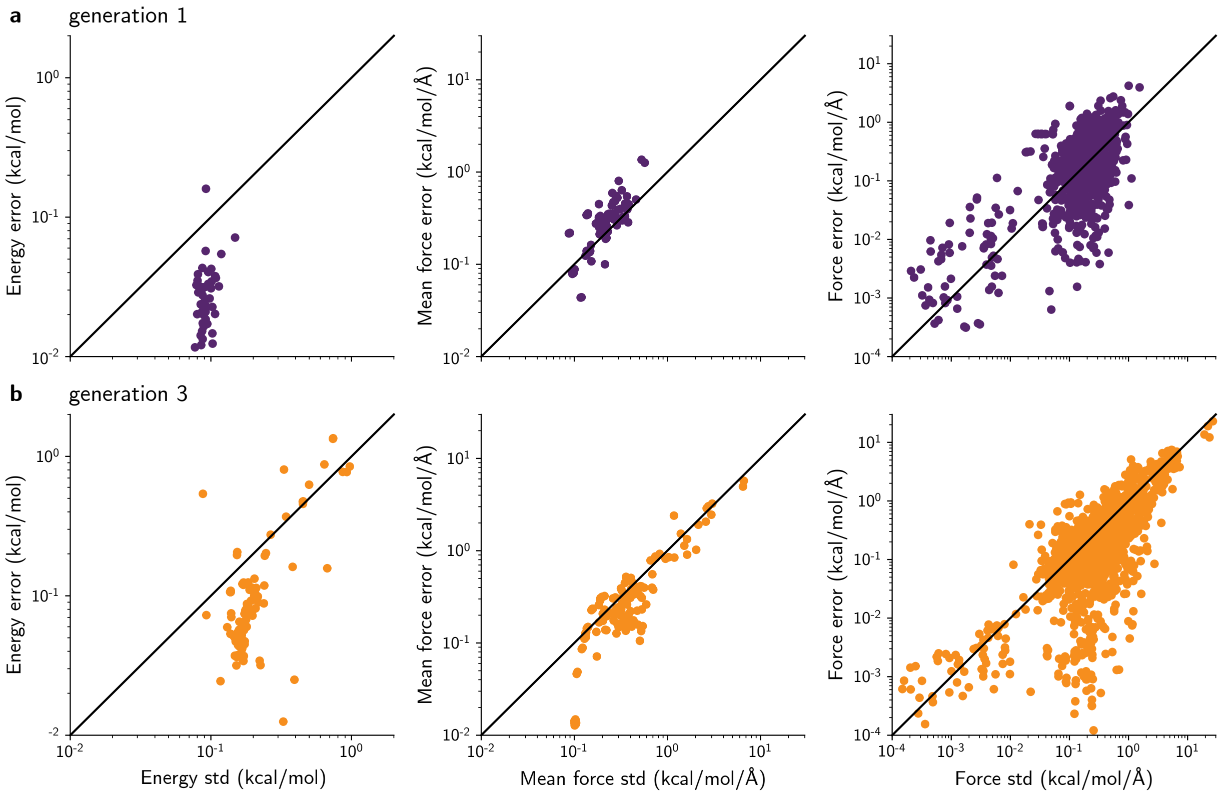

Supplementary Figure 9. Relationship between the energy/forces uncertainty and error for **a**, first and **b**, third generation NN potentials trained with the adversarial sampling strategy. The standard deviation (std) of energies, mean force of atoms, and forces in individual atoms is computed with an ensemble of 5 NNs.

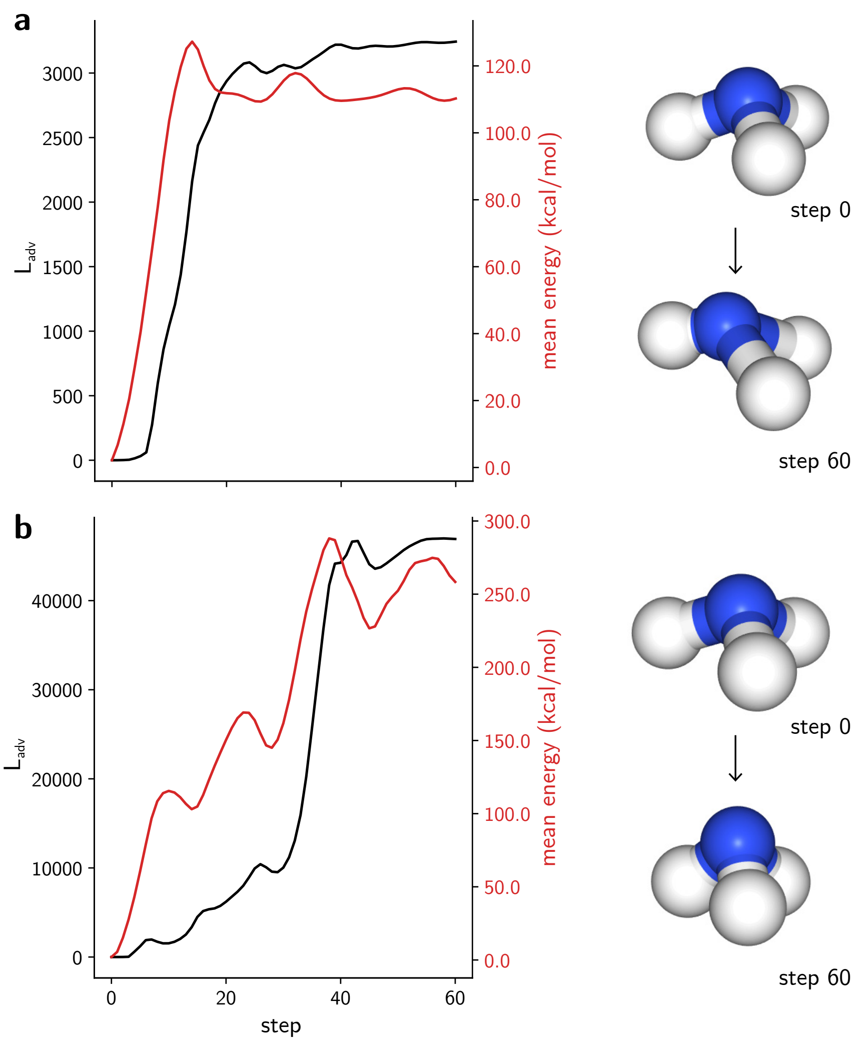

Supplementary Figure 10. Examples of an adversarial attack on the ammonia molecule using the third generation of NN potentials for this system. Within 60 optimization steps, the adversarial loss converges to higher energy geometries that include **a**, off-center distortions and **b**, symmetrically approaching the hydrogen atoms. The high energies are allowed by the adversarial loss due to a high sampling temperature ($kT = 20$ kcal/mol).

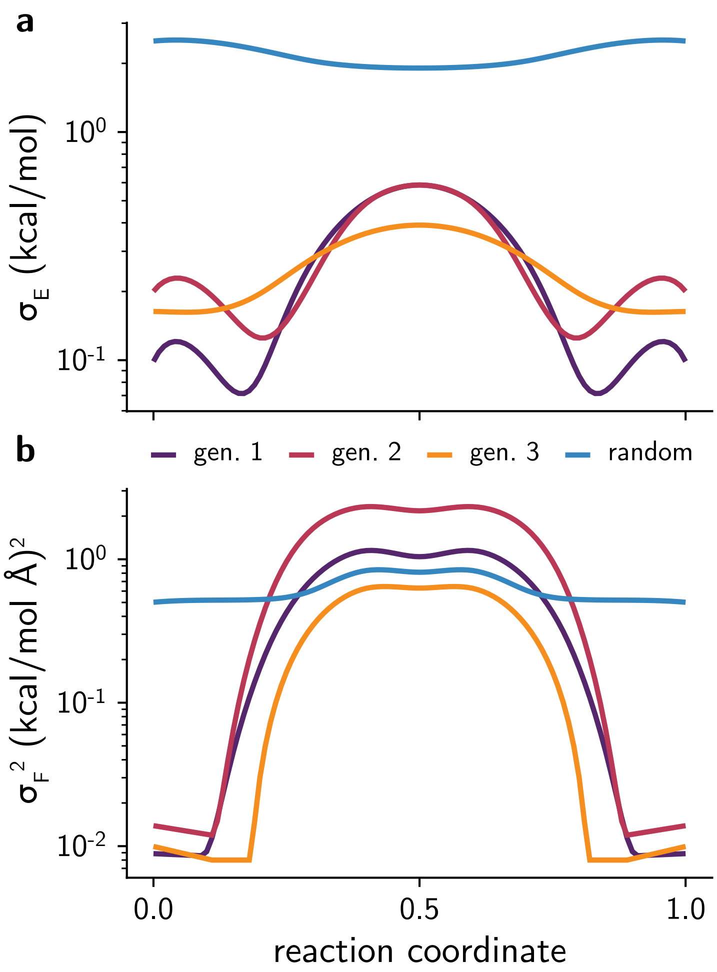

Supplementary Figure 11. Uncertainty in **a**, energy and **b**, forces for the nitrogen inversion barrier. Whereas the uncertainty close to the barrier tends to lower as the active learning loop progresses, it stays approximately constant for the NN potential trained on randomly-distorted geometries.

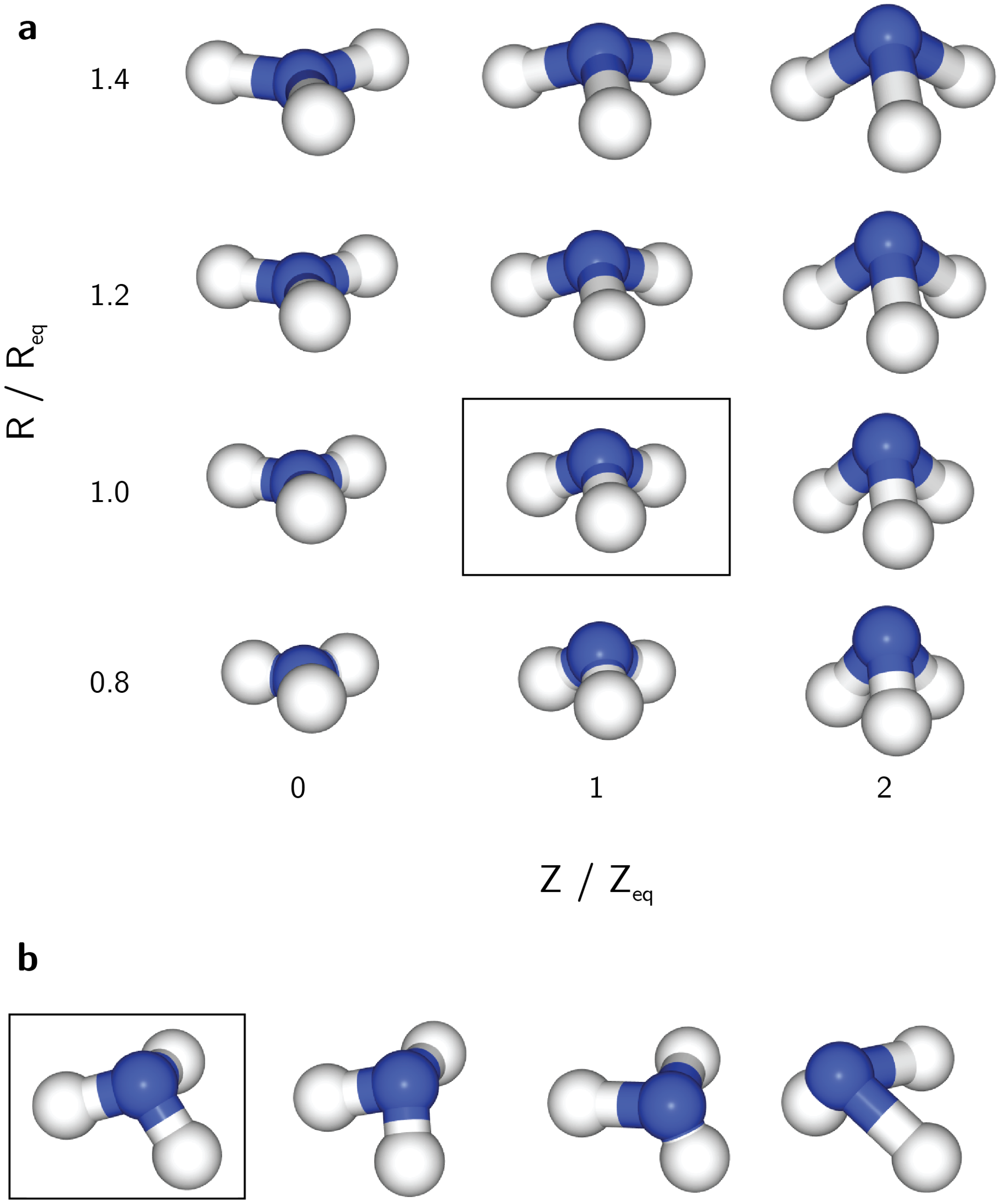

Supplementary Figure 12. **a**, Example of ammonia geometries created for different values of (Z, R). **b**, Different distortions of the ground state geometry (outlined in black) that have the same values of (Z_{eq}, R_{eq})

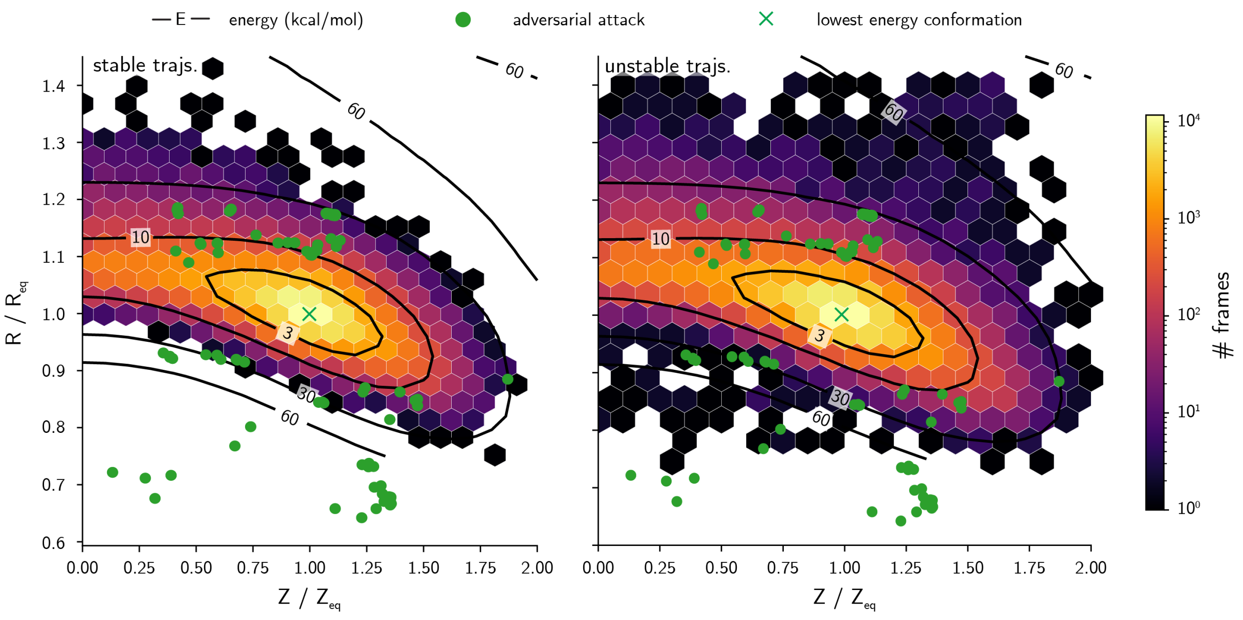

Supplementary Figure 13. Density of frames for stable (left) and unstable (right) molecular dynamics trajectories obtained with the third generation of NN potentials for ammonia. Contour lines indicate constant energy levels in the phase space. Points for 100 adversarial attacks performed for the third generation indicate that the adversarial strategy samples points not well explored by the NN potential in MD trajectories. Only a subset of the phase space of unstable trajectories is shown, as several data points fall outside of this region.

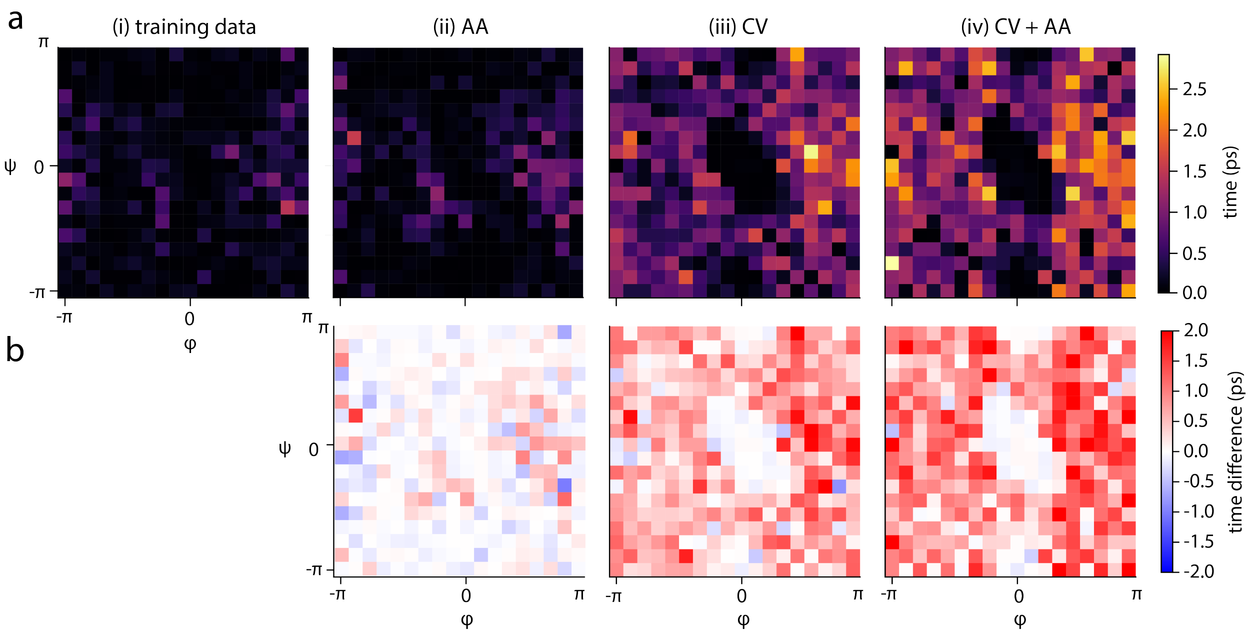

Supplementary Figure 14. **a**, Duration of stable molecular dynamics trajectories of alanine dipeptide, where grid points correspond to dihedral angles (φ, ψ) of starting configurations. MD trajectories are obtained with NN potentials trained on (i) only 10,000 MD data points, (ii) MD data + 3 generations of all-atom (AA) adversarial samples, (iii) MD data + 7 generations of predefined collective variable (CV) attacks, and (iv) MD data + 7 generations of CV attacks followed by 3 generations of AA attacks. **b**, Difference in durations of stable trajectories with respect to (i). Adding CV and AA attacks increases the robustness of NN potentials, allowing longer trajectories to be sampled.

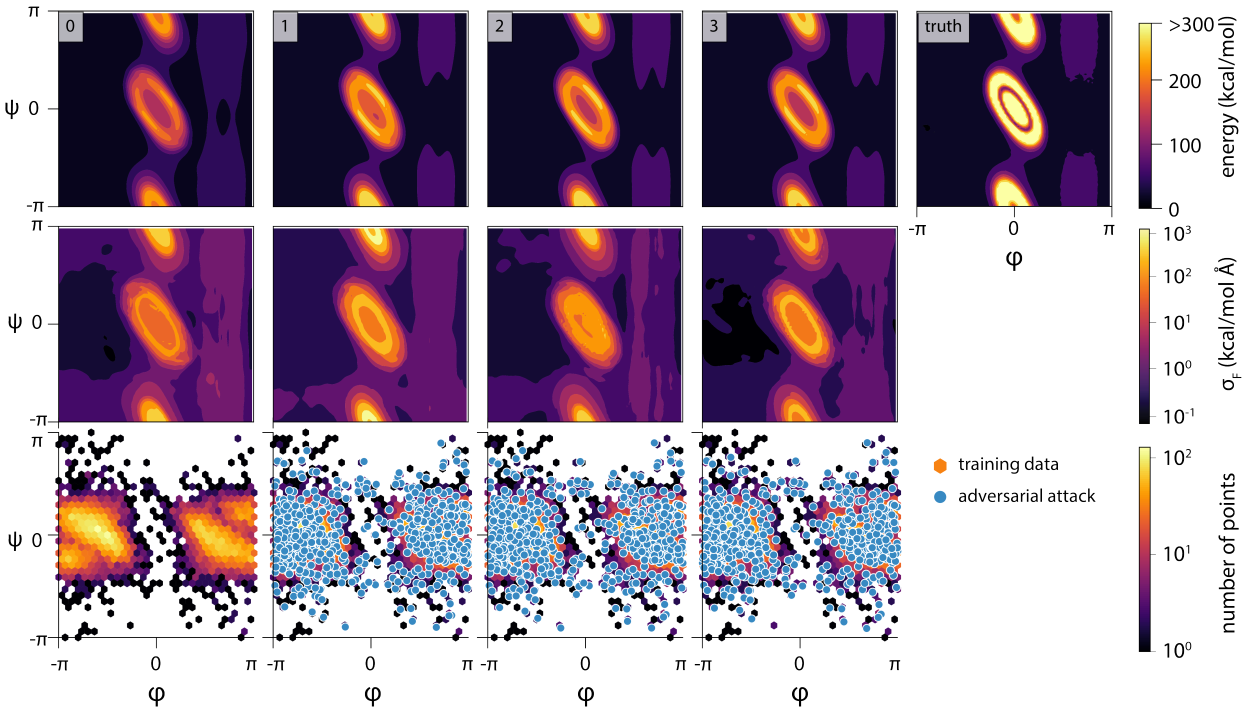

Supplementary Figure 15. Evolution of PES of an NN committee trained on 3 generations of adversarial examples. Adversarial examples (red points) are sampled by applying small displacements δ to positions of all atoms (AA). Generation 0 correspond to the same NN committee from generation 7 in the main paper (Fig. 4), where the committee was already trained on configurations from original MD training data and CV adversarial examples (all included as hexagonal bins in figure). Angles are given in radians.

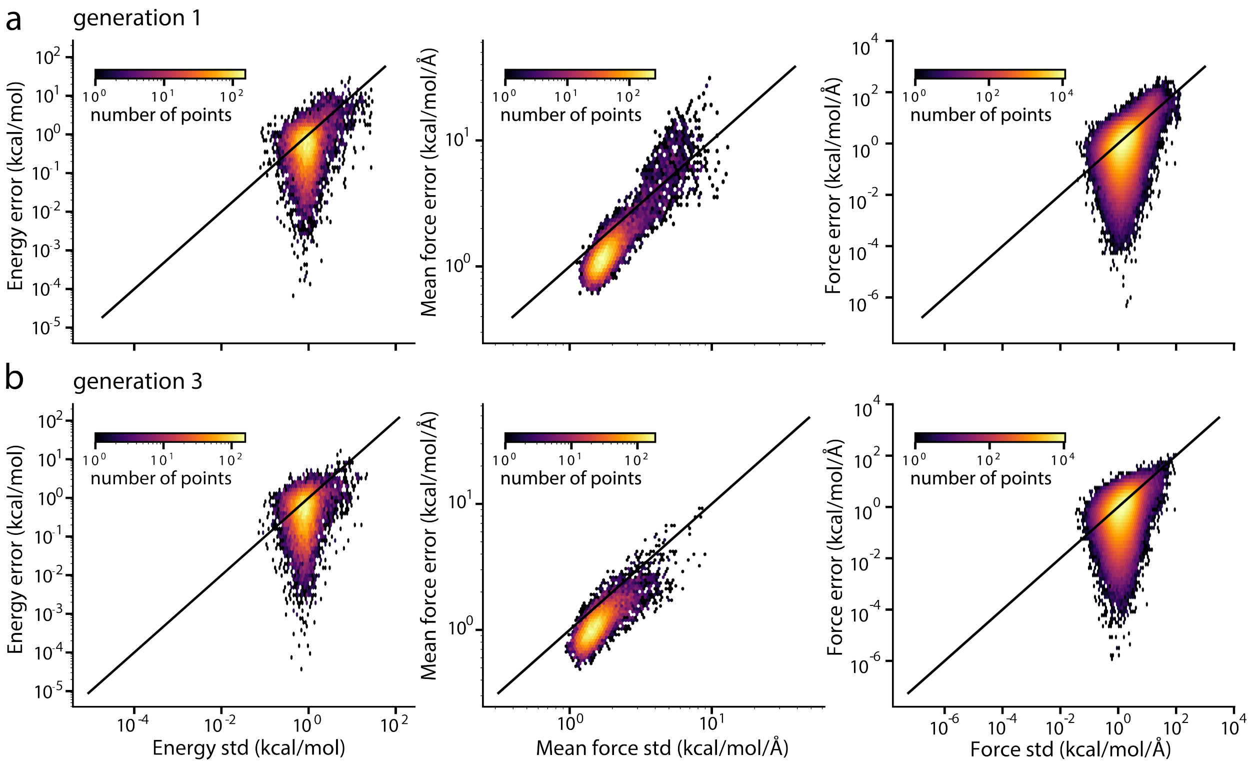

Supplementary Figure 16. Relationship between the energy/forces uncertainty and mean absolute error for **a**, first and **b**, third generation NN potentials trained on all-atom adversarial samples of alanine dipeptide. The NNs have been trained on the original MD dataset and 7 generations of predefined collective variable attack configurations (see Section III.C and Methods of the main paper). The standard deviation (std) of energies, mean force of atoms, and forces in individual atoms is computed with an ensemble of 5 NNs.

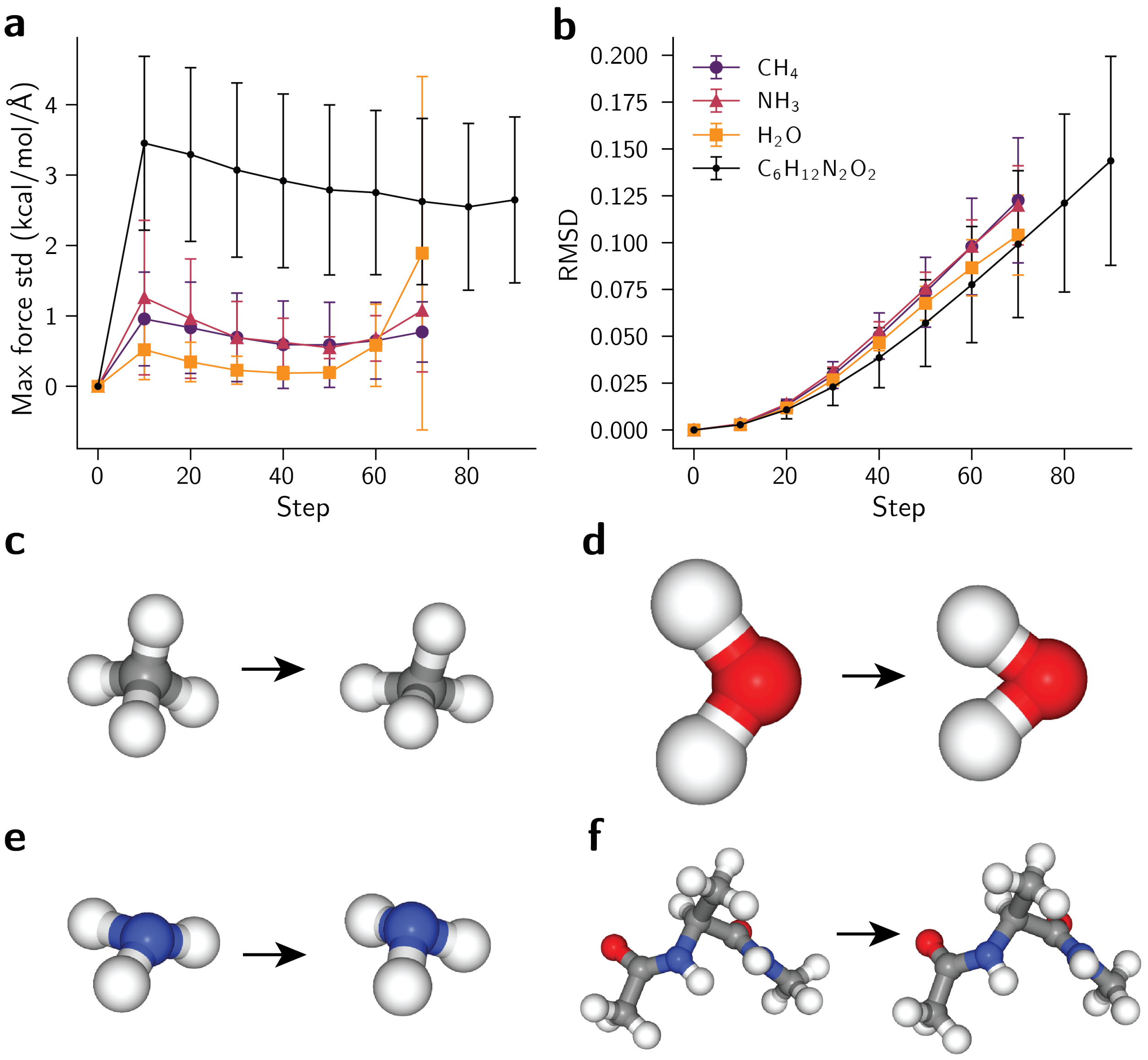

Supplementary Figure 17. Evolution of **a**, maximum standard deviation (std) of atomic forces, and **b**, RMSD between attacked geometry and initial seed as a function of adversarial steps. The adversarial attacks are performed on methane (CH_4), ammonia (NH_3), water (H_2O) and alanine dipeptide ($C_6H_{12}N_2O_2$) using ANI-1x models. Adversarial attacks are performed for 70 steps for small molecules and 100 steps for alanine dipeptide. Examples of configurations with high RMSD with respect to the initial seeds are shown in **c**-**f**.

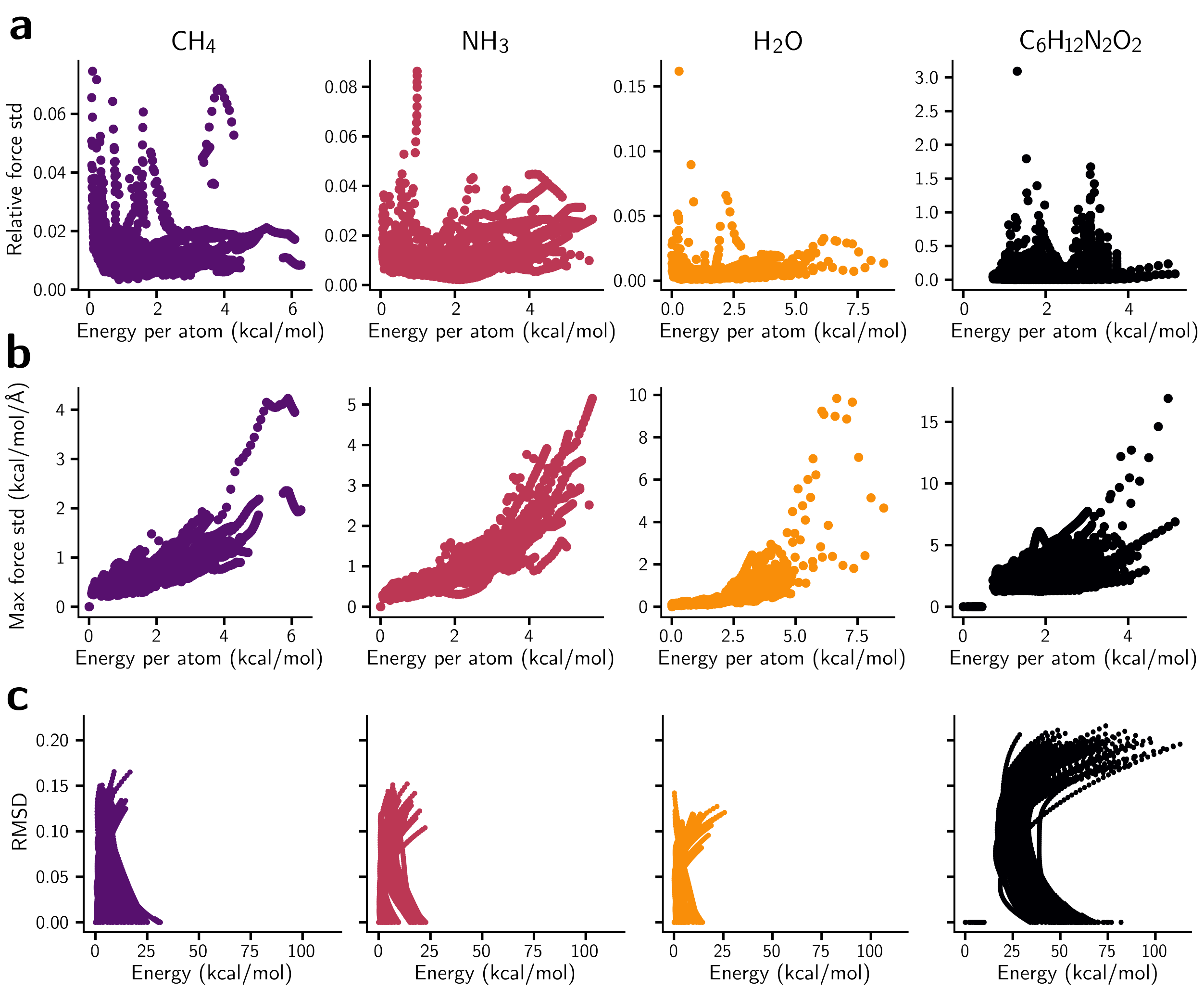

Supplementary Figure 18. **a**, Relative standard deviation (std) of atomic forces for all configurations obtained throughout the adversarial attacks on ANI-1x models. The relative force std is calculated according to Eq. (3). **b**, Maximum std of atomic force across models against energy per atom. **c**, RMSD of all configurations obtained during the adversarial attacks as a function of total energy of system. Adversarial attacks are performed using ANI-1x models for 70 steps for methane (CH_4), ammonia (NH_3), water (H_2O), and 100 steps for alanine dipeptide ($C_6H_{12}N_2O_2$).

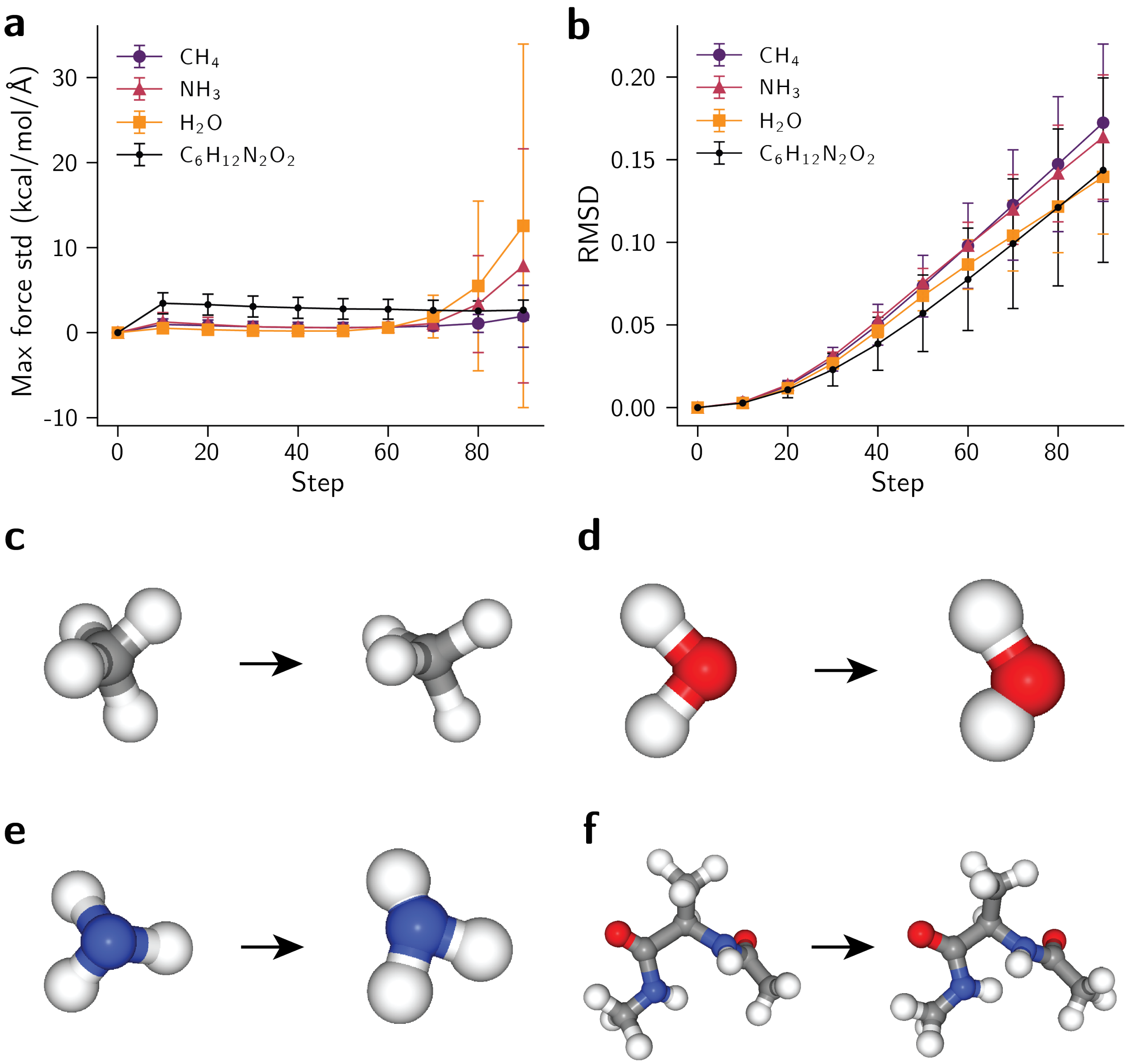

Supplementary Figure 19. Evolution of **a**, maximum standard deviation (std) of atomic forces, and **b**, RMSD between attacked geometry and initial seed as a function of adversarial steps. The adversarial attacks are performed on methane (CH_4), ammonia (NH_3), water (H_2O) and alanine dipeptide ($C_6H_{12}N_2O_2$) using ANI-1x models. Adversarial attacks are performed for 100 steps, where examples of configurations with high RMSD with respect to the initial seeds are shown in **c**-**f**.

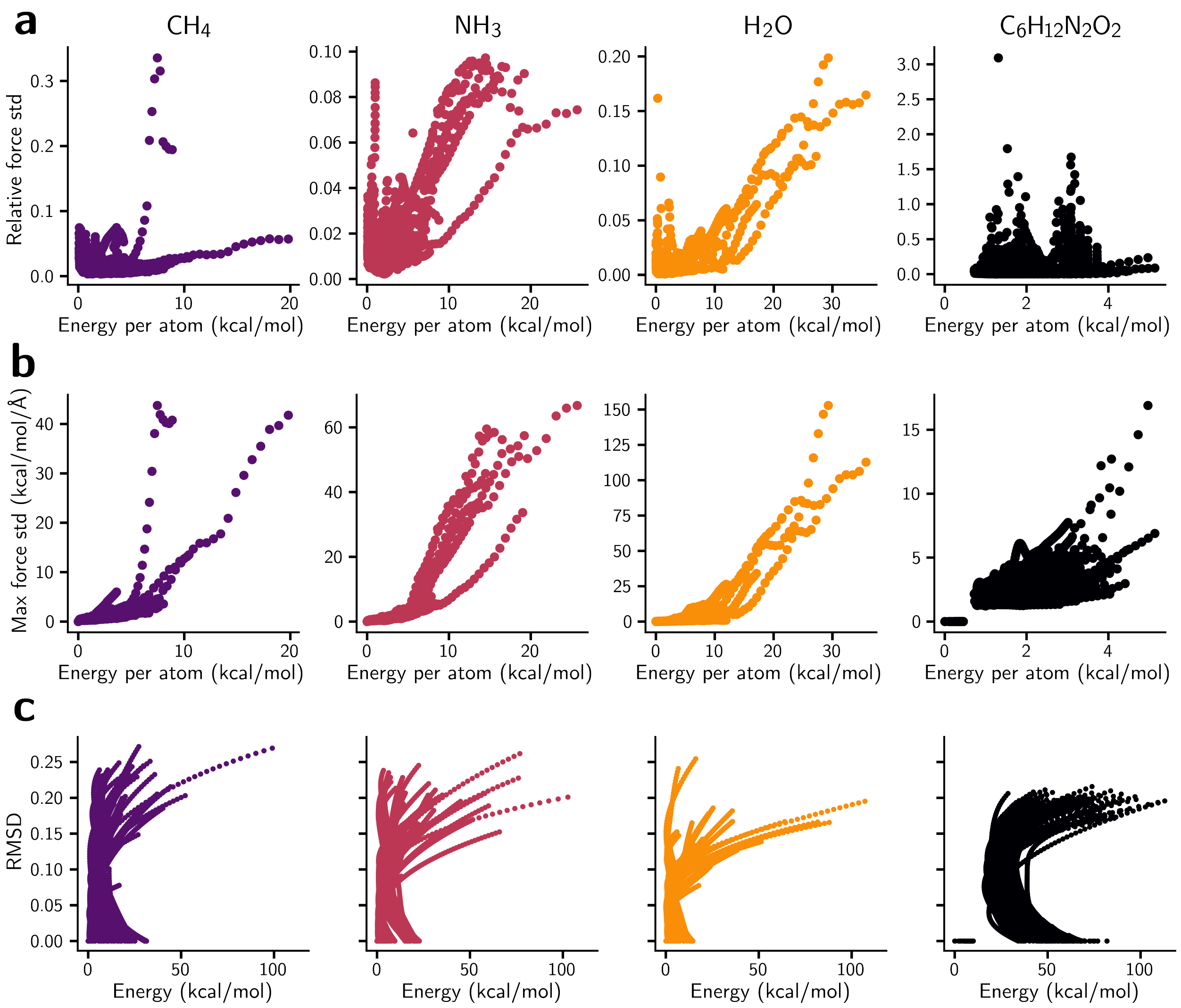

Supplementary Figure 20. **a**, Relative standard deviation (std) of atomic forces for all configurations obtained throughout the adversarial attacks on ANI-1x models. The relative force std is calculated according to Eq. (3). **b**, Maximum std of atomic force across models against energy per atom. **c**, RMSD of all configurations obtained during the adversarial attacks as a function of total energy of system. Adversarial attacks are performed using ANI-1x models for 100 steps for methane (CH_4), ammonia (NH_3), water (H_2O), and alanine dipeptide ($C_6H_{12}N_2O_2$).

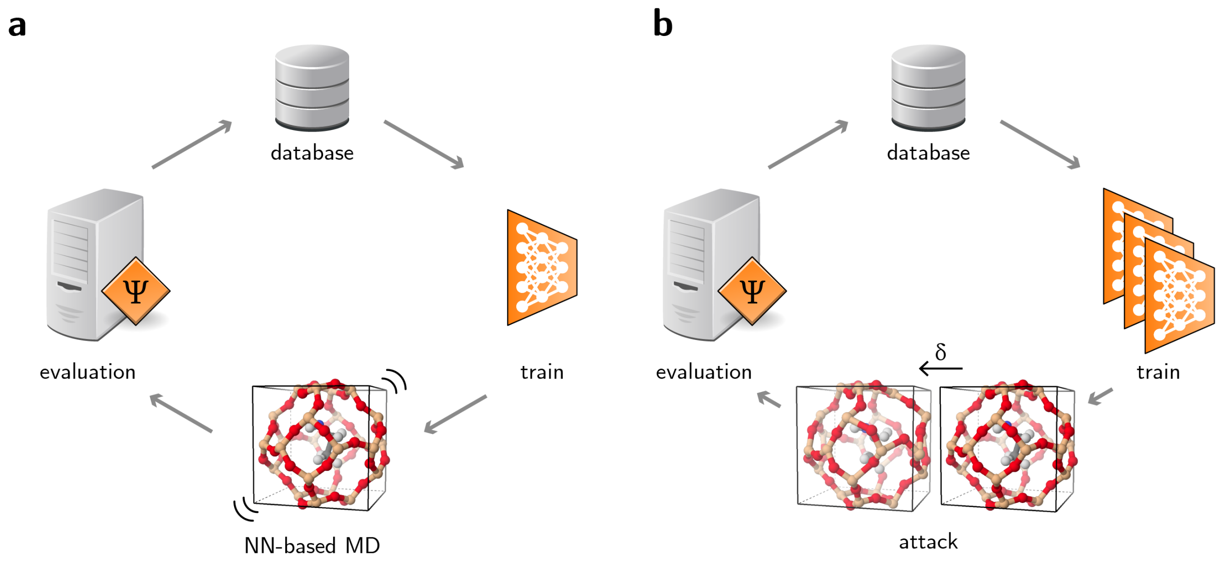

Supplementary Figure 21. Workflows for the active learning loops under study for our example on zeolites. **a**, Conventional strategy for improving neural network force fields using molecular dynamics simulations, and **b**, active learning loop using adversarial attacks to sample new geometries.

II. SUPPLEMENTARY TABLES

Supplementary Table 1. RMSD between attacked geometries against geometries within ANI-1x data set (training). Both the minimum and the maximum RMSDs are reported. Only methane (CH_4), ammonia (NH_3), and water (H_2O) molecules are compared.

RMSD (10^{-3})	H_2O	NH_3	CH_4
Min of attack vs training (70 steps)	2.7	9.7	4.1
Min of attack vs training (100 steps)	5.5	11.3	23.0
Max within training	334	372	408

Supplementary Table 2: k-points mesh for each of the zeolites studied in this work. All meshes were constructed using a uniform k-point density of 64 k-points/Å^{-3}.

Host	k-points mesh	Host	k-points mesh	Host	k-points mesh
ABW	$2 \times 4 \times 2$	ACO	$2 \times 2 \times 2$	AEI	$1 \times 1 \times 1$
AEL	$3 \times 1 \times 1$	AFI	$1 \times 1 \times 3$	AFN	$1 \times 1 \times 2$
AFR	$1 \times 1 \times 3$	AFY	$2 \times 2 \times 3$	APC	$2 \times 1 \times 2$
AST	$1 \times 1 \times 1$	ASV	$2 \times 2 \times 1$	ATN	$1 \times 1 \times 4$
ATS	$1 \times 1 \times 4$	AWW	$1 \times 1 \times 3$	BEC	$1 \times 1 \times 1$
BOF	$3 \times 1 \times 1$	BPH	$2 \times 2 \times 2$	CAN	$2 \times 2 \times 5$
CHA	$1 \times 1 \times 1$	CSV	$1 \times 2 \times 2$	CZP	$2 \times 2 \times 1$
DFT	$3 \times 3 \times 2$	DOH	$1 \times 1 \times 2$	EAB	$1 \times 1 \times 1$
GIS	$2 \times 2 \times 2$	GME	$1 \times 1 \times 2$	GON	$1 \times 1 \times 5$
JNT	$3 \times 1 \times 1$	JOZ	$3 \times 1 \times 1$	JRY	$3 \times 2 \times 1$
JSN	$2 \times 3 \times 1$	LAU	$1 \times 1 \times 3$	LOS	$2 \times 2 \times 2$
LTA	$2 \times 2 \times 2$	MEP	$1 \times 1 \times 1$	MER	$1 \times 1 \times 2$
MRE	$3 \times 1 \times 1$	MSO	$1 \times 1 \times 1$	MTT	$4 \times 1 \times 2$
MTW	$1 \times 4 \times 2$	MVY	$5 \times 3 \times 1$	MWW	$1 \times 1 \times 1$
NAT	$1 \times 1 \times 3$	OFF	$2 \times 2 \times 3$	OSO	$2 \times 2 \times 3$
OWE	$1 \times 3 \times 2$	PHI	$2 \times 1 \times 1$	PON	$2 \times 2 \times 1$

Continued on the next page

Host	k-points mesh	Host	k-points mesh	Host	k-points mesh
RRO	$3 \times 2 \times 1$	RTE	$1 \times 1 \times 3$	RTH	$2 \times 1 \times 2$
SAS	$1 \times 1 \times 2$	SAV	$1 \times 1 \times 2$	SBN	$3 \times 3 \times 1$
SFE	$2 \times 4 \times 1$	SFF	$2 \times 1 \times 3$	SFN	$1 \times 4 \times 1$
SFO	$1 \times 1 \times 3$	SOD	$2 \times 2 \times 2$	SOS	$1 \times 3 \times 2$
SSY	$4 \times 1 \times 1$	THO	$1 \times 3 \times 3$	TON	$1 \times 1 \times 4$
VET	$1 \times 1 \times 5$	YUG	$2 \times 1 \times 3$		

Supplementary Table 3: Zeolites and molecules studied in this work. The guests are identified by their SMILES string. Different poses have a different unique identifier (ID). All poses are available along with Supplementary Information of this paper and at `https://github.com/learningmatter-mit/Atomistic-Adversarial-Attacks`.

ID	Host	Guest SMILES	Guests/Cell
84712237	ABW	C1CN2CCN1CC2	1
84765818	ABW	C1CN2CCN1CC2	1
84753482	ABW	C1CN2CCN1CC2	1
84737015	ACO	NCCN	1
84767595	ACO	NCCN	2
85003055	ACO	NCCN	3
87082779	ACO	NCCN	3
90598419	AEI	C[C@H]1CCC[C@H](C)N1C	3
90656769	AEL	CC(C)NC(C)C	1
84848250	AFI	C1CCNCC1	1
88948280	AFI	C1CN2CCN1CC2	1
84810683	AFI	C1CN2CCN1CC2	1
84812164	AFI	C1CNCCN1	1
84926511	AFI	C1CNCCNC1	1
84761226	AFI	C1CNCCNC1	1
84966882	AFI	C1COCCN1	1

Continued on the next page

ID	Host	Guest SMILES	Guests/Cell
86513841	AFI	C1COCCN1	1
84765258	AFI	CC(C)(C)CN	1
84747569	AFI	CC(C)(C)CN	2
84765563	AFI	CC(C)(C)CN	2
84703582	AFI	CC(C)N	1
84747987	AFI	CC(C)N	1
84771834	AFI	CC1(C)CCCC(C)(C)N1	1
84947896	AFI	CCCN	1
85104383	AFI	CCN(CC)CC	1
89234486	AFI	CN1CCNCC1	1
84736439	AFI	CN1CCNCC1	1
86628511	AFI	CN1CCNCC1	2
84767357	AFI	CNC	1
84704458	AFI	CNC	1
84809582	AFI	CNC	3
84731622	AFI	CNC	3
84743714	AFI	C[C@@H](CN)CCCN	1
84752207	AFI	C[C@H](CN)CCCN	1
84780683	AFI	C[C@H](CN)CCCN	1
89133792	AFI	C[C@H]1CCC[C@H](C)N1	1
85380621	AFI	Cc1ccc(Cc2nnn[nH]2)cc1	1
84846686	AFI	Cc1nccn1C	1
84764779	AFI	NCCCCCCN	1
87257507	AFI	NCCCCCCN	1
84767598	AFI	NCCCCCCN	1
86522728	AFI	NCCN	1
84948309	AFI	NCCN	2
86105697	AFI	NCCNCCN	1

Continued on the next page

ID	Host	Guest SMILES	Guests/Cell
84700226	AFI	N[C@H]1CC[C@H](N)CC1	1
84718908	AFI	Nc1ccc(F)cc1	1
85137729	AFI	Nc1ccccn1	1
84762706	AFI	c1ccc(CN2CCCCC2)cc1	1
86966261	AFN	C1CNCCN1	4
84823581	AFN	CC(C)N	2
87807474	AFN	CC(C)N	5
88234760	AFN	C[C@H](O)CN(C[C@H](C)O)C[C@H](C)O	1
84797907	AFN	NCCCN	1
84816058	AFN	O=C1NCCCN1	1
85697486	AFN	O=C1NCCCN1	1
85629166	AFR	C=CNC=C	1
86520952	AFR	Nc1ccccc1	1
85812363	AFR	Nc1ccccc1	1
84797756	AFR	c1ccc2[nH]ccc2c1	1
84878530	AFR	c1ccc2[nH]ccc2c1	1
85006374	AFR	c1ccc2[nH]ccc2c1	1
84866500	AFR	c1ccc2ncccc2c1	1
90722319	AFR	c1ccncc1	2
84756248	AFY	CCCCN	1
84766894	AFY	CCCCN	1
84745105	AFY	CCCCN	1
87638176	AFY	CCCNCCC	1
84735981	AFY	CCCNCCC	1
84753454	AFY	CCCNCCC	1
84753458	AFY	CCN(CC)CC	1
84772451	AFY	CCN(CC)CC	1
84751962	AFY	CCN(CC)CC	1

Continued on the next page

ID	Host	Guest SMILES	Guests/Cell
88241668	AFY	CCN(CC)CC	2
87182781	AFY	NCCN	1
84695623	AFY	NCCN	1
84746623	AFY	NCCN	1
84751178	AFY	NCCN	2
84769681	AFY	NCCN	2
85402945	APC	CCCNCCC	1
90664509	AST	C1CN2CCC1CC2	3
90483624	AST	C1CN2CCC1CC2	4
90650122	AST	C1C[C@H]2CC[C@@H]1CNC2	4
90484877	AST	CC(C)(C)N	4
84743372	ASV	CNC	1
84758941	ASV	CNC	1
84690924	ASV	CNC	1
84806560	ASV	CNC	2
84741901	ATN	CCN(CC)CC	1
85221338	ATN	CCN(CC)CC	2
84770604	ATS	CC(C)NC(C)C	1
84794243	ATS	CC(C)NC(C)C	2
84741973	ATS	CCN(C(C)C)C(C)C	1
85809301	AWW	C1CCCNCC1	1
84943939	BEC	C1CN2CCN1CC2	4
84754315	BEC	CN(C)CCN(C)C	1
84888780	BEC	CN(C)CCN(C)C	2
84822087	BEC	CN1C[C@@H]2[C@H](C1)[C@@H]1C=C[C@H]2N(C)C1	1
85493354	BEC	CN1C[C@H]2[C@H]3C=C[C@@H]([C@H]2C1)N(C)C3	1
84801714	BEC	CN1C[C@H]2[C@H]3C=C[C@@H]([C@H]2C1)N(C)C3	1
84809064	BOF	C1CNCCN1	2

Continued on the next page

ID	Host	Guest SMILES	Guests/Cell
86545653	BOF	C1CNCCN1	3
86636213	BOF	C1CNCCN1	3
86541341	BOF	C1CNCCN1	4
86497676	BOF	CNC	1
85493350	BOF	CNC	1
84825368	BOF	CNC	2
84797755	BOF	NCCCN	2
85358360	BOF	NCCCN	3
84845077	BOF	NCCN	1
84693903	BOF	NCCN	1
84773059	BOF	NCCN	3
84941645	BOF	NCCN	3
87250311	BPH	CCN(CC)CC	1
85280332	BPH	CCN(CC)CC	2
89290369	CAN	C1CNCCN1	2
84781594	CAN	CCCN	1
84758465	CAN	CCCN	1
84766095	CAN	CCCN	1
84746813	CAN	CCCN	2
84719736	CAN	CCCN	2
90400997	CAN	CCN	2
84700228	CAN	CCN(CC)CC	1
89371135	CAN	CN1CCCC1	2
90452058	CAN	NC1CC1	2
90449760	CAN	NC1CC1	3
84765817	CAN	NCCCCCCN	1
90549916	CHA	C1CCNC1	2
90090428	CHA	C1CCNC1	3

Continued on the next page

ID	Host	Guest SMILES	Guests/Cell
90090424	CHA	C1CCNCC1	2
90450646	CHA	C1CN2CCN1CC2	2
90199762	CHA	C1CNCCN1	2
90456838	CHA	C1COCCN1	2
90429547	CHA	CC(C)NC(C)C	2
90506833	CHA	C[C@@H]1CNC[C@H](C)C1	2
90834283	CHA	C[N+](C)(C)[O-]	3
85781222	CSV	C1CCC(N2CCCCC2)CC1	1
84751797	CSV	C1CCC(N2CCCCC2)CC1	1
86571286	CZP	NCCNCCNCCN	1
84898924	CZP	NCCNCCNCCN	1
84786139	DFT	C1CNCCN1	1
84708533	DFT	C1CNCCN1	1
84751796	DFT	CNC	1
84730342	DFT	CNC	1
84749147	DFT	CNC	1
84755391	DFT	NCCCCN	1
84696267	DFT	NCCCCN	1
84715864	DFT	NCCCN	1
84758427	DFT	NCCCN	1
84753455	DFT	NCCN	1
84744838	DFT	NCCN	1
90813337	DOH	C1CN2CCC1CC2	6
90808097	EAB	C1COCCN1	3
88905648	EAB	CCC(N)CC	3
90548485	EAB	CCCN	4
89234633	EAB	CCN(C)C	4
90597397	EAB	CCP(CC)CC	3

Continued on the next page

ID	Host	Guest SMILES	Guests/Cell
90596216	EAB	C[C@@H]1CNC[C@H](C)C1	3
90790440	EAB	NC1CCCCC1	4
84749420	EAB	NCCCCCCN	4
90539869	EAB	NCCN	3
84749954	GIS	C1CNCCN1	1
84765968	GIS	C1CNCCN1	1
84856962	GIS	C1CNCCN1	2
84936354	GIS	C1CNCCN1	2
84770024	GIS	CC(C)N	1
84765593	GIS	CC(C)N	1
84767596	GIS	CC(C)N	1
85707048	GIS	CC(C)N	2
84751956	GIS	CC(C)NC(C)C	1
84792032	GIS	CC(C)NC(C)C	1
84778466	GIS	CCCNCCC	1
84761756	GIS	CNC	1
84738583	GIS	CNC	1
85280333	GIS	CNC	3
84752298	GIS	C[C@H](N)CN	1
84773363	GIS	C[C@H](N)CN	1
86544985	GIS	C[C@H](N)CN	2
85224874	GIS	C[C@H](N)CN	3
88903607	GIS	C[C@H](N)CN	3
84947938	GIS	C[C@H](N)CN	4
84743713	GIS	Cc1nc2ccccc2[nH]1	1
84739435	GIS	NCCCCCCN	1
88900683	GIS	NCCCCCCN	1
87259689	GIS	NCCCCCCN	1

Continued on the next page

ID	Host	Guest SMILES	Guests/Cell
84734462	GIS	NCCCCN	1
84741903	GIS	NCCCCN	1
84734465	GIS	NCCCCN	1
84771111	GIS	NCCCCN	2
84736680	GIS	NCCN	1
84750276	GIS	NCCN	2
84740540	GIS	NCCN	2
84758430	GIS	NCCN	2
86776503	GIS	NCCN	4
88241940	GIS	NCCNCCNCCN	1
84771153	GIS	c1ccc2[nH]cnc2c1	1
84745103	GIS	c1ccc2[nH]cnc2c1	1
84936924	GIS	c1ccc2[nH]cnc2c1	3
84813896	GME	C1CN2CCN1CC2	1
84814387	GME	C1CN2CCN1CC2	1
85714105	GME	C1CN2CCN1CC2	2
84753453	GME	C1CN2CCN1CC2	3
86531164	GME	C1CNCCN1	1
86899299	GME	C1CNCCN1	1
90515838	GME	CCNC	5
84743916	GME	CCP(CC)CC	1
84741894	GME	CCP(CC)CC	1
84720515	GME	CCP(CC)CC	1
88047668	GME	CCP(CC)CC	2
84747988	GME	CCP(CC)CC	2
84751298	GME	CCP(CC)CC	2
90551108	GME	CN(C)C	4
90481177	GME	CN(C)C	4

Continued on the next page

ID	Host	Guest SMILES	Guests/Cell
90599606	GME	C[C@@H]1CNC[C@H](C)C1	2
90672280	GME	Cc1ncc[nH]1	3
90629372	GME	NC1CCCCC1	3
84751302	GME	NCCNCCNCCN	1
84744991	GME	NCCNCCNCCN	1
84752297	GME	NCCNCCNCCN	1
90526917	GME	c1ccncc1	3
85707049	GON	C1CN2CCN1CC2	1
85864996	JNT	CN1CCNCC1	1
85851237	JNT	CN1CCNCC1	2
86344693	JOZ	C1CN2CCN1CC2	1
84920511	JOZ	C1CN2CCN1CC2	1
84888779	JOZ	C1CN2CCN1CC2	2
85632448	JOZ	C1CN2CCN1CC2	2
84740538	JOZ	C1CN2CCN1CC2	3
85851238	JOZ	C1CN2CCN1CC2	4
84849641	JRY	CCNCC	1
85978944	JRY	CCNCC	2
87735410	JSN	CCNCC	1
84735983	JSN	CCNCC	1
84715414	JSN	CCNCC	1
85142696	JSN	CCNCC	2
84765592	JSN	CCNCC	2
86520953	LAU	C1CCNC1	1
84823353	LAU	C1CCNC1	1
85714257	LAU	C1CCNC1	2
86661944	LAU	C1CN2CCC1CC2	2
86045265	LAU	C1CN2CCN1CC2	1

Continued on the next page

ID	Host	Guest SMILES	Guests/Cell
86477967	LAU	C1CN2CCN1CC2	1
85009875	LAU	c1ccncc1	1
90571098	LOS	CCNC	3
84805526	LTA	C1CCCNCC1	1
85829420	LTA	C1CCCNCCC1	1
85202111	LTA	C1CNCCN1	1
86161031	LTA	C1CNCCN1	2
84874656	LTA	C1CNCCNC1	2
85396384	LTA	C1CNCCNC1	2
89234207	LTA	C1COCCN1	1
84934527	LTA	C1COCCN1	2
88904745	LTA	C1COCCOCCNCCOCCOCCN1	1
85348139	LTA	C1COCCOCCOCCN1	1
85115723	LTA	C1COCCOCCOCCN1	1
90273387	LTA	C1COCCOCCOCCOCCOCCN1	1
88899990	LTA	C1COCCOCCOCCOCCOCCN1	1
84715225	LTA	C1N2CN3CN1CN(C2)C3	1
84749421	LTA	C1N2CN3CN1CN(C2)C3	1
84852420	LTA	C1N2CN3CN1CN(C2)C3	2
84735242	LTA	NC1CCCCC1	1
88904894	LTA	NC1CCCCC1	3
84716937	LTA	NC1CCCCCCC1	1
84811154	LTA	OCCN(CCO)CCO	1
84945347	LTA	OCCN(CCO)CCO	1
90644347	MEP	CCC[C@@H](C)N	7
84926701	MER	CC1(C)C[C@@H]2C[C@@](C)(CN2)C1	1
85370027	MER	CC1(C)C[C@@H]2C[C@@](C)(CN2)C1	2
89729159	MRE	CCN	1

Continued on the next page

ID	Host	Guest SMILES	Guests/Cell
90568573	MSO	C1COCCOCCOCCOCCO1	2
85714172	MTT	C1CCCNCC1	2
84831102	MTT	CC(C)(C)N	2
84886784	MTT	CC(C)CN	2
85179178	MTT	CC(C)N	1
88898522	MTT	CC(C)N	1
87253495	MTT	CC(C)N	2
85029109	MTT	CC(C)N	2
84812455	MTT	CC(C)NC(C)C	1
86428540	MTT	CC(C)NCCCN	1
87182803	MTT	CCN	2
84877108	MTT	CCNCCC(C)C	2
85357316	MTT	CCNCCN(C)C	1
84947087	MTT	NCCCN	1
84939714	MTW	C1CN2CCC1CC2	2
88190817	MTW	CC(C)CN	2
84708499	MVY	CC(C)N	1
84705545	MVY	CC(C)N	1
84778465	MVY	CC(C)N	2
84762375	MVY	CCN	1
84712239	MVY	CCN	1
84741975	MVY	CCN	2
84755392	MVY	CCN	2
84738966	MWW	NCCN	1
84773795	MWW	NCCN	1
84766069	MWW	NCCN	1
89879561	MWW	NCCN	8
90073525	MWW	NCCN	9

Continued on the next page

ID	Host	Guest SMILES	Guests/Cell
88904144	MWW	NCCN	11
85649016	NAT	C1CN2CCN1CC2	1
85816736	NAT	C1CN2CCN1CC2	2
86513645	NAT	C1CN2CCN1CC2	3
84743712	NAT	C1CN2CCN1CC2	4
84751957	NAT	NCCN	1
84758466	NAT	NCCN	1
85656279	NAT	NCCN	2
86572462	NAT	NCCN	3
86648377	NAT	NCCN	4
90412714	OFF	C1CCNCC1	2
90258222	OFF	C1CN2CCN1CC2	2
90531115	OFF	C1CNCCN1	2
89533074	OFF	CCCN	2
89291998	OFF	CCN(C)C	2
90037855	OFF	CN(C)C	2
90568572	OFF	CN(C)C1CCCCC1	2
90650312	OFF	CP(C)C	2
89437811	OFF	c1ccncc1	3
84749796	OSO	NCCNCCN	1
84720490	OSO	NCCNCCN	1
84761227	OWE	CC(C)(N)CN	1
84769495	OWE	C[C@@H](N)CN	2
84741898	OWE	C[C@H](N)CN	1
84759526	OWE	C[C@H](N)CN	1
84708749	OWE	C[C@H](N)CN	2
85851235	PHI	C1CCNCC1	1
84931018	PHI	C1CN2CCN1CC2	1

Continued on the next page

ID	Host	Guest SMILES	Guests/Cell
85957094	PHI	C1CN2CCN1CC2	1
84946009	PHI	C1CN2CCN1CC2	1
84809581	PHI	C1CN2CCN1CC2	2
86161034	PHI	C1CNCCN1	5
85001545	PHI	C1CNCCN1	6
87378812	PHI	C1CNCCN1	7
85593627	PHI	C1COCCN1	1
86501174	PHI	C1COCCN1	1
88903050	PHI	CN1CCN(C)CC1	1
85011401	PHI	CN1CCN(C)CC1	2
85654333	PHI	CN1CCN(C)CC1	2
85694555	PHI	c1c[nH]cn1	2
85261277	PON	CN	1
84738840	PON	CN	3
84741240	RRO	CN	1
84750766	RRO	CN	1
84767602	RRO	CN	1
84697643	RRO	CN	2
84788641	RRO	CN	2
86541344	RRO	CN	3
86541343	RRO	CN	3
84767001	RRO	CNC	1
84705343	RRO	CNC	1
84700227	RRO	CNC	1
86595136	RRO	CNC	2
88182711	RRO	CNC	2
86329513	RTE	C1C[C@H]2CC[C@@H]1CNC2	1
84815465	RTE	C1C[C@H]2CC[C@@H]1CNC2	2

Continued on the next page

ID	Host	Guest SMILES	Guests/Cell
86678261	RTE	C1C[C@H]2CC[C@@H]1CNC2	2
84732584	RTE	CN1C(C)(C)CCCC1(C)C	1
84761753	RTE	CN1C(C)(C)CCCC1(C)C	1
84740237	RTE	CN1C(C)(C)CCCC1(C)C	1
84799129	RTE	CN1C(C)(C)CCCC1(C)C	2
84754316	RTE	CN1C(C)(C)CCCC1(C)C	2
86550735	RTE	N[C@@H]1C[C@H]2CC[C@@H]1C2	1
87898573	RTH	C1CCCNCC1	1
86680528	RTH	C1CCCNCC1	1
85599546	RTH	C1CCCNCC1	1
85203091	RTH	C1CCCNCC1	2
85710631	RTH	C1CCCNCC1	3
85419932	RTH	C1CCNCC1	1
84941644	RTH	C1CCNCC1	1
84937356	RTH	C1CCNCC1	1
84814797	RTH	C1CNCCN1	2
86595099	RTH	C1CNCCN1	2
84739794	RTH	CC(C)NC(C)C	1
84733549	RTH	CC(C)NC(C)C	1
84740542	RTH	CC(C)NC(C)C	1
84807974	RTH	CC(C)NC(C)C	2
84776633	RTH	CC(C)NC(C)C	2
84816060	RTH	CC(C)NC(C)C	2
88900148	RTH	CC(C)NC(C)C	3
88237619	RTH	CC(C)NC(C)C	3
84940646	RTH	CC(C)P(C(C)C)C(C)C	2
86945739	RTH	CC1CCNCC1	1
85243835	RTH	CC1CCNCC1	1

Continued on the next page

ID	Host	Guest SMILES	Guests/Cell
85824362	RTH	CN1CCCCC1	1
86532185	RTH	CN1CCCCC1	1
85015051	RTH	CN1CCCCC1	2
84887262	RTH	CN1CCCCC1	2
84749800	RTH	CN1CCCCC1	2
85788592	RTH	C[C@@H]1CNC[C@H](C)C1	1
86259035	RTH	C[C@@H]1CNC[C@H](C)C1	2
84891355	RTH	C[C@H]1CNC[C@H](C)C1	2
84796597	RTH	Cc1nc(C)n(C)c1C	1
86648457	RTH	Cc1nc(C)n(C)c1C	1
88902609	RTH	Cc1nc(C)n(C)c1C	2
84939713	RTH	Cc1nccn1C	1
85853686	RTH	Cc1nccn1C	1
86541340	RTH	Cc1nccn1C	2
85090894	RTH	Cc1nccn1C	2
84866012	RTH	Cc1nccn1C	4
84939429	RTH	N[C@H]1C[C@@H]2CC[C@H]1C2	2
85492991	RTH	N[C@H]1C[C@H]2CC[C@@H]1C2	1
86538902	RTH	N[C@H]1C[C@H]2CC[C@@H]1C2	2
84935879	RTH	c1ccncc1	1
86560006	RTH	c1ccncc1	2
84815466	SAS	C1CCNCC1	1
84909278	SAS	C1CCNCC1	1
84812802	SAS	C1CCNCC1	1
87844739	SAS	C1CCNCC1	2
86636241	SAS	CC1(C)CCCC(C)(C)N1	2
84797309	SAS	CCN	1
86544147	SAS	CCN	1

Continued on the next page

ID	Host	Guest SMILES	Guests/Cell
84813467	SAS	CCN	1
85458594	SAS	CCN	2
84919326	SAS	CCN	2
84902972	SAS	CN1CCCN(C)CCN(C)CCCN(C)CC1	1
84944525	SAS	CN1CCCN(C)CCN(C)CCCN(C)CC1	1
85051707	SAS	NCCN	1
84802621	SAS	NCCN	1
84897820	SAS	NCCN	2
90367036	SAV	CCN(C(C)C)C(C)C	3
87220809	SBN	CN	1
84717919	SBN	CN	1
84764780	SBN	CN	1
84696265	SBN	CN	2
84758013	SBN	CN	2
84699687	SBN	CN	3
84735982	SBN	CN	3
84704213	SBN	CN	3
84718677	SBN	NCCNCCN	1
84758014	SBN	NCCNCCN	1
84739436	SBN	c1ccncc1	1
84773321	SBN	c1ccncc1	1
84719737	SBN	c1ccncc1	1
84752208	SBN	c1ccncc1	2
84747257	SBN	c1ccncc1	2
84741899	SBN	c1ccncc1	2
84750108	SBN	c1ccncc1	3
84702940	SFE	C1CC[C@@H]2NCCC[C@@H]2C1	1
84757059	SFE	C1CC[C@H]2NCCC[C@H]2C1	1

Continued on the next page

ID	Host	Guest SMILES	Guests/Cell
84749952	SFE	CN(C)c1ccncc1	1
84769496	SFE	CN(C)c1ccncc1	1
84765119	SFE	CN(C)c1ccncc1	1
84751301	SFF	CCN1[C@@H](C)CCC[C@@H]1C	1
84762923	SFF	CCN1[C@@H](C)CCC[C@H]1C	1
84763335	SFF	CCN1[C@@H](C)CCC[C@H]1C	1
88900452	SFF	CCN1[C@@H](C)CCC[C@H]1C	2
84856727	SFF	CCN1[C@H](C)CCC[C@H]1C	2
84775176	SFN	C1CCCNCCC1	1
88906451	SFN	C1CCCNCCC1	1
86148119	SFN	CCN(CC)CC	1
87797279	SFO	CN(C)c1ccncc1	1
87651313	SFO	CN(C)c1ccncc1	1
84747873	SFO	CN(C)c1ccncc1	1
84734466	SFO	CN(C)c1ccncc1	2
84741467	SOD	C1CCNC1	1
84771631	SOD	C1CCNC1	1
84749799	SOD	C1CCNC1	1
84708922	SOD	C1CN2CCN1CC2	1
84748186	SOD	C1CN2CCN1CC2	1
84703282	SOD	C1CN2CCN1CC2	1
89067194	SOD	C1CN2CCN1CC2	2
89768285	SOD	C1N2CN3CN1CN(C2)C3	1
89234459	SOD	CC(C)NC(C)C	1
90381331	SOD	CCC(N)CC	1
90253337	SOD	CCC(N)CC	2
84761225	SOD	CCN	1
84787804	SOD	CCN	1

Continued on the next page

ID	Host	Guest SMILES	Guests/Cell
84771630	SOD	CCN	1
84767000	SOD	CCN	2
84713063	SOD	CCN	2
84748438	SOD	CCN	2
90499837	SOD	CCN(C(C)C)C(C)C	1
84740720	SOD	CCN(CC)CC	1
84762922	SOD	CCN(CC)CC	1
84761755	SOD	CCN(CC)CC	1
86560003	SOD	CCN(CC)CC	2
89824077	SOD	CN(C)C	2
89251359	SOD	CN(C)C1CCCCC1	1
90562949	SOD	CN1CCCC1	1
89887547	SOD	CP(C)C	1
90558607	SOD	CP(C)C	2
90633114	SOD	C[C@@H]1CNC[C@H](C)C1	1
87254755	SOD	Cc1ncc[nH]1	1
88093264	SOD	Cc1ncc[nH]1	1
84737522	SOD	Cc1ncc[nH]1	2
84767640	SOD	Cc1ncc[nH]1	2
84737017	SOD	Cc1ncc[nH]1	2
84738837	SOD	NCCN	1
84751300	SOD	NCCN	1
87579034	SOD	NCCN	2
87292944	SOD	NCCN	2
90664510	SOD	NCCNCCN	1
90430223	SOD	N[C@]12C[C@H]3C[C@H](C[C@H](C3)C1)C2	1
90017479	SOD	N[C@]12C[C@H]3C[C@H](C[C@H](C3)C1)C2	2
84701287	SOD	c1c[nH]cn1	1

Continued on the next page

ID	Host	Guest SMILES	Guests/Cell
84750278	SOD	c1c[nH]cn1	1
84732944	SOD	c1c[nH]cn1	2
84742968	SOD	c1c[nH]cn1	2
84770282	SOD	c1ccncc1	1
84735400	SOD	c1ccncc1	1
84753181	SOD	c1ccncc1	1
84765564	SOD	c1ccncc1	2
84736437	SOD	c1ccncc1	2
88042729	SOD	c1cnc2c(c1)ccc1cccnc12	1
85003552	SOS	NCCNCCN	1
84866236	SOS	NCCNCCN	2
84890153	SOS	NCCNCCN	2
88230200	SSY	C1CCNC1	1
84743715	SSY	C1CCNC1	1
84773056	SSY	C1CCNC1	2
84852120	SSY	C1CCNC1	2
84704459	THO	C[C@@H](N)CN	1
84754738	THO	C[C@@H](N)CN	2
84746810	THO	C[C@@H](N)CN	2
84762223	THO	C[C@H](N)CN	1
84730493	THO	C[C@H](N)CN	1
84734463	THO	NCCCN	1
84744330	THO	NCCCN	1
84740236	THO	NCCCN	1
84743917	THO	NCCCN	2
84767643	THO	NCCCN	2
84761889	THO	NCCN	1
84706965	THO	NCCN	1

Continued on the next page

ID	Host	Guest SMILES	Guests/Cell
87729592	THO	NCCN	1
84767645	THO	NCCN	2
86963426	TON	CC(C)(C)CN	1
85215229	TON	CC(C)(C)CN	1
84809782	TON	CC(C)(C)CN	2
85134532	TON	CC1(C)CCCC(C)(C)N1	1
84877854	TON	CC1(C)CNCC(C)(C)C1	2
85005213	TON	C[C@@H]1CNC[C@H](C)C1	1
87581074	TON	NCCCCCCN	1
87844765	TON	NCCCCCCN	2
85670029	TON	c1ccncc1	1
85254981	VET	C1CC2(CCN1)OCCO2	1
84743370	VET	C[C@@H]1CCOC2(CCNCC2)O1	1
84756488	VET	NC1CCCC1	1
84744989	YUG	NCCCCCCN	1
84749423	YUG	NCCCCCCN	1
84743914	YUG	NCCCCCCN	1
84756798	YUG	NCCCCN	1
84767638	YUG	NCCCCN	1
84941848	ZON	C1CN2CCN1CC2	2
84930372	ZON	c1c[nH]cn1	1
85723716	ZON	c1c[nH]cn1	1
86661965	ZON	c1c[nH]cn1	4
